# Supplementary material for: Fractional response analysis reveals logarithmic cytokine responses in cellular populations
Source: Nat Commun. 2021 Jul 7;12:4175. doi: 10.1038/s41467-021-24449-2 (PMC8263596; doi:10.1038/s41467-021-24449-2)
Supplement: Supplementary file 1 — Supplementary Information [file 41467_2021_24449_MOESM1_ESM.pdf]

Supplementary Information for

Fractional response analysis reveals logarithmic cytokine responses in cellular populations

Karol Nienałtowski, Rachel E. Rigby, Jarosław Walczak, Karolina E. Zakrzewska, Edyta Głów, Jan Rehwinkel, and Michał Komorowski

Correspondence to: m.komorowski@sysbiosig.org

This PDF file includes:

Supplementary Notes
Supplementary Methods
Supplementary References
Supplementary Tables: 1 - 2
Supplementary Figures: 1 - 17

Table of content

Supplementary Notes

Supplementary Note 1. Interpretation of FRA in terms of information-theory 3

Supplementary Note 2. Comparison with Shannon information 5

2.1 Basics of Shannon information 5

2.2 Advantages of Rényi min-information over Shannon information for cellular signaling 6

2.3 Quantitative relationship between Rényi min-information capacity and Shannon capacity 7

2.4 Comparison of Rényi min-information capacity and Shannon capacity based on GPCR signaling data 7

Supplementary Note 3. FRA can detect differences in responses masked by population averages 11

3.1 Discrimination between binary and graded response 11

3.2 Mean responses mask IFN-γ sensitivity in populations of cancer cells 12

Supplementary Note 4. Caveats of FRA 14

4.1 Sufficient data size is required for accurate estimation 14

4.2 Results might be dependent on the choice of doses 15

Supplementary Note 5. R-package 16

Supplementary Methods

1. Equivalency of Eq. 4 in the main paper and Eq. 11 17

2. Cancer cell lines methods (corresponding to section 3.2 of Supplementary Note 3) 17

Supplementary References 19

Supplementary Tables 20

Supplementary Figures 22

Supplementary note 1. Interpretation of FRA in terms of information-theory

The FRA is inspired by the mathematical theory of information, specifically the concepts of Rényi min-information[1–4]. Broadly speaking, one of the concerns of information theory is to quantify how much information about an input variable X is contained in an output variable Y. Typically, the input variable denotes signals (messages) that need to be decoded from the output variable[5–7]. To explain how the FRA relates to information theory, assume that doses, $x_1,\ldots,x_i$, represent signals that need to be decoded from the cellular responses, y, by an external observer. If response distributions to each dose were completely distinct, each of the i doses could be decoded by the observer without error, as responses to each dose correspond to a different range. On the other hand, if distributions corresponding to different doses exhibited some overlap, a decoding strategy would be needed to translate a response back to the dose. It is well established in statistics, the best decoding strategy is the intuitive one: assign the signaling response to the dose for which it is most frequent[5]. Then, among cells stimulated with the dose x_k, the faction that could be decoded correctly is the fraction that exhibits responses most frequent for x_k, which in the paper is referred to as typical and quantified as v_{kk} and is shown in Fig. 2f-i for the three doses example. FRC, $r(x_i)$, as shown in Eq. 11 of the main paper and derived in Supplementary Methods, can be written as the sum of fractions of cells that can be decoded correctly

$$r(x_i) = \sum_{k=1}^{i} v_{kk}, \tag{1}$$

The fact that $r(x_i)$ is the sum of fractions that can be decoded correctly implies that $\frac{1}{i}r(x_i)$ is the probability that an observer would guess correctly the dose of a randomly selected cell among cells stimulated with any of the i doses. Therefore, the product of the number of doses, i, and the probability of correct decoding $\frac{1}{i}r(x_i)$, which equals $r(x_i)$, can be interpreted as the number of different doses that can be decoded correctly on average.

The above intuitive reasoning, which provided the interpretation of $r(x_i)$ in terms of the number of different doses that can be decoded correctly on average by the observer, is formalized within information-theory by the Rényi min-information. Rényi min-information, similarly to Shannon's information, allows to quantify how much information about a variable X is transferred to a variable Y.

Precisely, for a set of input signals $x_1,...,x_i$ and output responses distributed as $P(Y|x_i)$ the information transferred from X to Y is quantified by Rényi min-information capacity, $C^*_{\min}$, defined as[1,3,4]

$$C^*_{\min} = \log_2 \int_y \max_{x_k \le x_i} P(y|x_k)dy\,. \tag{2}$$

$C^*_{\min}$ is expressed in bits of Rényi min-entropy[1,3,4,6] and can be interpreted as the $\log_2$ of the number of different messages that can be transferred from X to Y in the sense of Rényi min-entropy.

We have, therefore, that

$$C^*_{\min} = \log_2(r(x_i)) \tag{3}$$

or equivalently

$$r(x_i) = 2^{C^*_{\min}}\,, \tag{4}$$

which provides interpretation of FRC in terms of Rényi information capacity: $r(x_i)$ is the number of resolvable doses among $x_1,...,x_i$.

Supplementary Note 2. Comparison with Shannon information

2.1 Basics of Shannon information

Biochemical signaling has been traditionally analyzed in the framework of Shannon information[7–10]. Shannon information is derived from Shannon entropy, which in the context of biochemical signaling can be, perhaps, most simply introduced in the following way. Assume that input values, $x_1,\ldots,x_m$, follow a certain distribution, $P(X) = (P(x_1).,\ldots,P(x_m))$. Shannon entropy of $P(X)$ defined as

$$H(X) = -\sum_{i=1}^{m} P(x_i)\log_2(P(x_i)).$$

quantifies the uncertainty regarding the input values when no output is observed. When, on the other hand, output is observed, the probability that the input value x_i generated the output y is given by the Bayes formula

$$P(x_i|Y=y) = \frac{P(y|X=x_i)}{\sum_{j=1}^{m} P(y|X=x_j)},$$

and has associated Shannon entropy

$$H(X|Y=y) = -\sum_{i=1}^{m} P(x_i|Y=y)\log_2(P(x_i|Y=y)).$$

As the above entropy describes uncertainty regarding input value after observing a specific output value, y, and is not representative of all possible values of the output. Averaging $H(X|Y)$ over all output values, y , which are distributed as

$$P(y) = \sum_{i=1}^{m} P(y|X=x_i)P(x_i),$$

gives the average entropy of the input after observing the output

$$H(X|Y) = \int_{y} H(X|Y=y)P(y)dy.$$

The difference between initial, *a priori*, entropy of the input, $H(X)$, and average entropy of the input given the output, *a posteriori*, H(X|Y), quantifies the information gain that defines the Shannon information

$$I(X,Y) = H(X) - H(X|Y).$$

Shannon information depends on the input distribution, $P(X)$. Considering an input distribution that maximizes mutual information, referred to as optimal input distribution, $P^*(X)$, leads to maximal mutual information known as information capacity

$C^* = \max_{P(X)} I(X, Y)$.

Information capacity was defined by Shannon[5] to quantify $\log_2$ of the maximal number of discrete symbols that can be transferred in a single transmission with a negligible error when messages are encoded in terms long sequences of discrete symbols, through a communication channel described by the probability distribution $P(Y|X)$. Such interpretation of Shannon capacity is known as Shannon coding theorem[5].

Shannon information gained widespread applicability in engineering and has also been adapted to quantify the amount of information transfer in cellular signaling pathways. It can be debated, however, whether it is the most suitable measure for quantification of information flow along cellular signaling relays. For instance, Shannon information depends on the input distribution, $P(X)$, which in most applications is unknown, and a rationale for a choice of a particular distribution is missing. Besides, maximization with respect to the input distribution for the calculation of information capacity raises the question of whether cellular signaling actually operates under optimal conditions. Last but not least, cellular signaling systems do not transfer information with messages encoded in terms of long sequences of discreet symbols, which makes the interpretation of Shannon information difficult to formulate under the circumstances in which cellular signaling operates.

2.2 Advantages of Rényi min-information over Shannon information for cellular signaling

The Rényi min-capacity, C^*_{min}, does not suffer from the above drawbacks. It does not depend on the input distributions, and it does not involve long sequences of discrete symbols for rigorous interpretation. As explained before, (i) $2^{C^*_{min}}$ is the sum of fractions of cells simulated with a given dose with responses typical (most likely) for this dose, and (ii) $2^{C^*_{min}}/m$ is that the probability of a cell generating a response typical for a dose it was exposed to. If an external observer is decoding the input signals using the maximum likelihood principle, than $2^{C^*_{min}}$ is the sum of fractions of cells that can be decoded correctly, and $2^{C^*_{min}}/\mathrm{m}$ is the overall probability of correct decoding (see Supplementary Table 2

for the summary). In addition, Rényi min-capacity offers the decomposition into fractions of cells with different response levels, which can be presented along with FRC and as pie-charts.

2.3 Quantitative relationship between Rényi min-information capacity and Shannon capacity

The relationship between Shannon capacity and Rényi min-capacity is not well established under general conditions. Nevertheless, it has been shown that[1,4]

$$C^*_{\min} \geq C^*. \tag{5}$$

We computed $C^*_{\min}$ and C^* for CyTOF data, Supplementary Fig. 13a, as well as for IL-10, IFN-γ and TNF-α responses, Supplementary Fig. 13b. Shannon capacity was computed using R-package SLEMI[11]. The power of 2 of the Shannon information capacity, 2^{C^*}, ranged from 1.37 and 2.37 being smaller by 0.75 on average from Rényi measure $2^{C^*_{min}}$. The question, which quantification more adequately represents signaling performance of the studied systems cannot be debated without reference to the axiomatic definition of Rényi entropies. Nonetheless, in contrast to Shannon, Rényi information is straightforward to interpret, which is one of its main strengths.

2.4 Comparison of Rényi min-information capacity and Shannon capacity based on GPCR signaling data

For further comparison of the Rényi min-information capacity and Shannon information capacity, we considered a data set published in the *Keshelava et al. Nat Commun 2018*[12], where authors quantify information transfer by G protein-coupled receptor signaling.

Authors stimulated human kidney embryo cells, HEK293, with different doses of acetylcholine (Ach), which activated the muscarinic acetylcholine receptor M3R - the only member of the acetylcholine GPCR group expressed in HEK293 cells - resulting in Ca^{2+} intracellular influx, which was measured as the signaling output in individual cells. Individual cells were exposed 5 times to each of 7 increasing doses of Ach so that 35 measurements were taken for each cell so that individual quantitative characteristics of individual cells were obtained, examining 433 cells in total. For each dose, the data enabled the construction of response distributions both for the population of cells as well as for individual cells. Dose-response distributions of the population are shown as violin plots in

Supplementary Fig. 14a, whereas dose-responses of three individual cells are presented in Supplementary Fig. 14b.

The authors first used the population distributions, Supplementary Fig. 14a, to calculate Shannon information capacity. Here, calculation of Shannon capacity using R-package SLEMI[11] yielded 0.89 bits ($\log_2(1.85)$). Calculation of Shannon capacity for population distributions implicitly assumes that cell-to-cell heterogeneity constitutes a noise source leading to information loss. Therefore, subsequently, the authors calculated capacity using response distributions of individual cells, which quantified information transfer of individual cells, i.e., under noise arising inside of single-cells, and not from cell-to-cell heterogeneity. The average capacity among individual cells was 1.65 bits ($\log_2(3.14)$). The considerably higher capacity in the latter scenario indicated that not all of cell-to-cell heterogeneity can be interpreted as noise leading to information loss inside of individual cells. Individual cells might be in different states that determine their dose-response levels, and different cells in the population are in different states[12].

Here, for comparison Rényi min-information, embedded in FRA, with Shannon information, we first analyzed population response distributions, Supplementary Fig. 14c. The value of FRC for the highest dose, 2.03, indicates that overall cellular populations can generate two distinct distributions. Comparing FRC with Shannon capacity, we have that FRC is more than 2^{C^*}, which is in line with the inequality of Eq. 5. In addition to the value of Rényi min-information, FRA reveals the overlaps between distributions are substantial for low and high doses and moderate for intermediate doses. As pointed out by the authors of the paper presenting the data, Shannon information cannot be here interpreted as the capacity of cells to discriminate between doses due to cell-to-cell heterogeneity, which is not equivalent to noise. In contrast, the interpretation of Rényi min-information capacity presented above is valid, as it does not refer directly to the capacity of individual cells to discriminate between the doses. Furthermore, FRA is designed to quantify dose responses of a heterogeneous population of cells.

As the considered data set involves response distributions of individual cells, the question arises whether FRA could be deployed to analyze these in addition to population response distributions. From technical perspective, using single-cell response distributions, as opposed to population response distributions is irrelevant. FRA can be easily performed for each cell, nevertheless, interpretation of FRC and overlaps

between response distributions need to be established anew. By analogy with population response distributions, Supplementary Table 2, we describe two possible interpretations:

(i) an increase in FRC, $r(x_i)$ - $r(x_{i-1})$, can be interpreted as the probability of a given cell to generate a response to the dose x_i that is distinct from all lower doses (more likely for x_i than for any lower dose). The value of $r(x_i)$ quantifies that the overall number of distinct response distributions specific to an examined cell. Value of FRC for the maximal dose, $r(x_m)$, is the maximal number of distinct response distributions that a given cell generates. Overlaps between response distributions, as quantified by pie-charts, and color bands around FRC, show probabilities of a given cell stimulated with a given does to generate a response that is typical (most frequent) for any of the considered doses;

(ii) considering the best possible strategy to decode the dose from the output of individual cells, i.e., maximum likelihood decoding, FRA can be interpreted in terms of probabilities of correct and incorrect decoding. The increase in FRC shows probability of correctly discriminating the dose x_i from lower doses. Values of the FRC, $r(x_i)$, is the sum of probabilities of correct decoding of the doses not greater than x_i. Therefore, $r(x_m)$/m, is the overall probability of correct decoding. Finally, pie-charts, and color bands around FRC, show probabilities of decoding a response to a given dose (rows) as another dose (columns).

FRA plots for three representative individual cells are shown in Supplementary Fig. 14d. The highest value of FRC for individual cells is higher than for the population responses. An individual cell can generate, therefore, more distinct response distribution than a population of cells, which is in line with findings presented in *Keshelava et al. Nat Commun 2018*[12]. Analogously, an individual cell can resolve between higher numbers of doses than predicted by the population response distributions. In addition, FRA plots quantify that cells differ in terms of which concentrations can be resolved, some are better in resolving small doses, and other high doses.

For comparison of the extent to which different cells differ in terms of potential to discriminate between different doses we calculated, $r(x_m)$, which equivalent to $2^{C^*_{min}}$, for each cell and presented distribution of $2^{C^*_{min}}$, as histograms, Supplementary Fig. 14e. Besides, we computed analogous distribution using Shannon information capacity, i.e., distribution with 2^{C^*} with C^* calculated for each cell, Supplementary

Fig. 14f. Distributions corresponding to both types of approaches are similar with $2^{C^*_{min}}$ exhibiting higher vales, in line with inequality of Eq. 5. Again, both approaches yield similar conclusions, yet Shannon information capacity is less straightforward to interpret.

The above analysis implies several insightful conclusions regarding applicability of information theory to quantify information transfer in single-cells. As pointed out by *Keshelava et al. Nat Commun 2018*[12], responses of individual cells are much less variable than overall cell-to-cell heterogeneity of responses. Cell-to-cell heterogeneity cannot be interpreted as noise leading to information loss about the level of signaling input. Shannon information capacity, therefore, calculated based on population response distributions cannot be interpreted in terms of potency of individual cells to resolve between different concentrations. Each cell is different and therefore to calculate its potential to resolve between different concentrations its own response distribution should be determined. On the other hand, Rényi information can be calculated and meaningfully interpreted in terms of fractions of cellular populations. Calculation of Shannon information capacity based on distributions of individual cells can be done and interpreted to the extent provided by Shannon framework, including Shannon coding theorem. On the other hand, interpretation of Rényi is straightforward and does not require any advanced formalism like Shannon coding theorem.

Supplementary Note 3. FRA can detect differences in responses masked by population averages

In order to demonstrate how FRA can uncover patterns masked by population averages and, hence, discriminate between different response modalities, we considered two examples. An *in silico* model was tailored to generate graded and binary responses and illustrate how the two response modes are represented by FRA. In the second example, IFN-γ responses in two cancer cell lines were studied to demonstrate how mean responses might mask stimulation sensitivity in cancer cell populations, which can be rescued by FRA.

3.1 Discrimination between binary and graded response

In order to demonstrate how FRA can discriminate between graded and binary responses, we considered a synthetic model in which the same mean response results from different response distributions in cellular populations. Denote the response of a cell in the cellular population as Z, log of the response as $Y=\log_{10}(Z)$, and stimulation level as x. Assume that the mean log response, μ=E(Y), is described as

$$\mu(x) = \mu_{off} + (\mu_{on} - \mu_{off})\frac{x^2}{1+x^2}, \quad (6)$$

where μ_{off} denotes the mean baseline log-response, i.e., the off-state mean for x=0, and μ_{on} the maximal log-response, i.e., the on-state mean for saturating x, Supplementary Fig. 15a. The same mean can arise in different modes of stochastic responses, i.e., in graded or binary responses, Supplementary Fig. 15b,c. In the graded mode, the responses are centered around the mean, which assuming normal distribution of log-responses can be written as

$$P(Y|X = x) = N(\mu(x), \sigma^2), \quad (7)$$

where σ^2 denotes the log-response variance, Supplementary Fig. 15b. In the binary case, the log-responses can be assumed to center around the basal level, μ_{off}, or around the fully induced level, μ_{on}, which by further assumption of log-response normality gives two possible response distributions $N(\mu_{off}, \sigma^2)$, or $N(\mu_{on}, \sigma^2)$.

If the probability of a cell generating a response following the first distribution is given as $\frac{1}{1+x^2}$ and the second as $\frac{x^2}{1+x^2}$ then the overall probability of responses is given as

$$P(Y|X = x) = \frac{1}{1+x^2}N(\mu_{off}, \sigma^2) + \frac{x^2}{1+x^2}N(\mu_{on}, \sigma^2), \quad (8)$$

which is shown in Supplementary Fig. 15c and has the mean given by Eq. 6. Having constructed the model, we considered six representative doses, x=0, ½, 1, 1 ½, 2, and performed FRA, Supplementary Fig.15d-g.

For both cases, FRC shifts gradually, Supplementary Fig. 15d,e, however, for the graded system FRC reaches the value of over 3, compared to nearly 2 for the binary case. The maximal FRC value of 3 reflects the property of the considered graded system to generate responses in the range typical for basal, intermediate, and saturating doses. In contrast, the binary system can only generate responses in two ranges, basal and saturating, which leads to the maximal FRC value of 2. The structure of the responses of the two systems is further reflected in the bands around FRC and the pie-charts, Supplementary Fig. 15d-g. For the graded system, the bands and pie-charts indicate that response distributions corresponding to a given dose overlap with distributions corresponding to several other doses. For the binary system, on the other hand, responses to different doses overlap only with the distributions corresponding to the minimal and the highest dose, therefore, the bands have only two colors, violet, below the FRC, which corresponds to the minimal dose, and green, above the FRC, corresponding to the highest dose.

The two response modalities are represented differently within FRA, highlighting differences masked by the population average. Similar differences in functioning of a more complex system represented by multivariate data could not be detectable with a simple visual inspection as for simple univariate examples. In such cases, the different response modalities could be uncovered with FRA.

Having performed FRA of the exact binary system allows comparison with IL-10 responses analyzed in the main text. IL-10 responses have only certain characteristics of the binary mode. The band above the FRC in the binary system is missing for IL-10 responses. IL-10 distributions flatten over a broad range of responses as the dose increases, with a high number of stimulated cells having responses in the range characteristic for non-stimulated cells, which is mirrored by the broad bands below FRC.

3.2 Mean responses mask IFN-γ sensitivity in populations of cancer cells

In order to further demonstrate how FRA can uncover patterns masked by population averages, we performed IFN-γ dose-response experiments on two lung cancer cell lines: A549 and CALU1 (see Supplementary Methods). Similarly as in U937 cells in the main paper, nuclear fluorescence

corresponding to the phosphorylated form of STAT1 was measured. Population distributions are shown in Supplementary Fig. 16a, whereas means of responses (red line) and means of log-responses (black lines) are shown in Supplementary Fig. 16b. Both variants of the mean indicate that A549 cells exhibit stronger responses than CALU1 over the whole range of considered concentrations. Nevertheless, an inspection of response distributions indicates that A549 cells exhibit higher cell-to-cell heterogeneity, compared to CALU1, which leads to stronger overlaps between distributions corresponding to different doses. Therefore, even though the mean responses shift more strongly for A549 than CALU1, it does not imply that the responses distributions are more distinct. It is possible that for increasing doses, a smaller fraction of cells exhibits different responses for A549 than for CALU1 due to higher cell-to-cell heterogeneity. We have therefore performed FRA for both cell lines, Supplementary Fig. 16c,d. As opposed to analysis of means, the FRC increases more rapidly for CALU1 than for A549 cells. The maximal value for CALU1 cells is achieved for lower doses. However, both cell lines reach the same maximal value. The more rapid increase of FRC for CALU1 reflects the fact that even though the increments in means due to dose increase are smaller than for A549 cells, the lower cell-to-cell heterogeneity makes the response distributions more distinct. In terms of fractions of cells that exhibit different responses due to dose change CALU1 is more sensitive to IFN-γ than A549, which is masked by the analysis of population averages. Accounting for cell-to-cell heterogeneity is, therefore, an essential component of sensitivity of cellular populations, which is implemented within FRA.

Supplementary Note 4. Caveats of FRA

4.1 Sufficient data size is required for accurate estimation

In order to examine how estimation of FRA depends on the data size and data dimensionality, we have considered a simple test model, previously explored in [10], involving two possible input values, $X \in \{x_1, x_2\}$, and an output $Y \mid X = x_i$ described by a d-dimensional normal distribution with a diagonal covariance matrix, and mean vectors that differ only in the first dimension. Precisely,

$$Y|x_i \sim N(\mu_i, \Sigma),\; Y \in \mathcal{R}^d, \text{for } i=1,2, \tag{9}$$

$$\mu_1 = (0,0,\dots,0), \qquad \mu_2 = (2,0,\dots,0),$$

$$\Sigma = \begin{pmatrix} 1 & \cdots & 0 \\ \vdots & \ddots & \vdots \\ 0 & \cdots & 1 \end{pmatrix}.$$

To account for non-normal distributions, we have also considered the exponent of the above model, $Z = \exp(Y)$,
which resulted in log-normally distributed data. Further, we have considered three different dimensionalities, d=1, 10, 100, 1000, and performed estimation of the cumulative fraction of cells, $2^{C^*_{min}}$, for different sample sizes, N, for the normal and log-normal data, Supplementary Fig. 17a,b. For comparison, we have calculated the true value via numerical integration.

For each d the estimation is accurate if the data size, N, is sufficiently large. In other words, the estimates converge to the true values with data size. For one-dimensional data, d=1, 4 measurements already appear to give relatively accurate estimates. For d=10, d=100 and d=1000, 32 and 256, 1024, measurements, respectively, are required. For insufficient N the values have a positive bias (are overestimated), which is to be expected. If data are too sparsely sampled, collected data points corresponding to different doses are likely to be distinct even though the actual distributions are not. In Supplementary Fig. 17c we summarize the above analysis by plotting minimal N required to ensure estimation with the precision of 1, 5, and 10% as a function of d. Overall, the above simple models

suggest that accurate estimation requires the number of cells to tangibly exceed the number of measured response variables.

Many, if not most, of the current high-throughput technologies provide measurements of hundreds, rather than tens, of cells per sample. In those cases, FRA should provide reliable analysis even for highly-dimensional data as long as hundreds of cells per sample are available. However, in scenarios in which thousands of response variables are measured per cell (e.g., different transcripts in scRNA-seq or highly multiplexed FISH), hundreds of cells may not suffice for accurate estimation. In such cases, either more cells per dose should be measured, or analysis should be limited to a smaller number of essential responsive genes.

4.2 Results might be dependent on the choice of doses

FRA depends on the number and distribution of doses over a relevant concentration range, which should accounted for when interpreting FRA results. Although interpretation of FRA is valid regardless of the selection of doses, for accurate representation of a signaling system doses should cover relevant concentration range in which a signaling system is sensitive to concentration changes. This is particularly important if an analysis is aimed to conclude about certain properties of a signaling system, rather than of a particular data set, for instance, about the possible number of programmed response distributions a given signaling system can generate. To illustrate how the number of doses impacts FRA, we have considered the graded model introduced in Section 3.1, Eq. 8. We varied the different number of doses ranging from 2 to 64. For each number of doses, the doses used for estimation included 0 and 10, whereas the other doses were equally distant in-between. The result of the estimation is shown in Supplementary Fig. 17d. The cumulative fraction of cells increases with the number of used doses. The difference in estimation for 4 and 64 doses is approximately 0.5, which appears to be relatively low. Nonetheless, FRA depends on the experimental data design, which should be taken into account when interpreting its results.

Supplementary Note 5. R-package

FRA implementation as R-package is available for download at http://github.com/sysbiosig/FRA.

After installation, as described in the deposited user manual:

https://github.com/sysbiosig/FRA/blob/master/Manual.pdf,

FRA can be performed in the following way. The input data should be available as a data-frame, here named `data`,

	input	output 1	output 2	…	output d
n_1	x_1	$y_1^{1,1}$	$y_1^{1,2}$		$y_1^{1,d}$
	$\vdots$	$\vdots$	$\vdots$	$\cdots$	$\vdots$
	x_1	$y_1^{n_1,1}$	$y_1^{n_1,2}$		$y_1^{n_1,d}$
n_2	x_2	$y_2^{1,1}$	$y_2^{1,2}$		$y_2^{1,d}$
	$\vdots$	$\vdots$	$\vdots$	$\cdots$	$\vdots$
	x_2	$y_2^{n_2,1}$	$y_2^{n_2,2}$		$y_2^{n_2,d}$
$\vdots$		$\vdots$	$\vdots$	$\ddots$	$\vdots$
n_m	x_m	$y_m^{1,1}$	$y_m^{1,2}$		$y_m^{1,d}$
	$\vdots$	$\vdots$	$\vdots$	$\cdots$	$\vdots$
	x_m	$y_m^{n_m,1}$	$y_m^{n_m,2}$		$y_m^{n_m,d}$

where rows correspond to individual cells. The first column "input" contains stimuli levels, x_i, whereas subsequent columns represent multivariate responses of individual cells, $y_i^{l,k}$, where l varies from 1 to n_i ,i.e., the number of cells measured for x_i, and k varies from 1 to d, i.e., the number of response covariates. Once the data-frame is available the model for computing FRA needs to defined, which in the simplest instance is done as follows

```
model=FRA::FRA( data= data,
                 signal=”input”,
                 response=c(”output_1”, ”output_2”, …,”output_d”)
                 )
```

FRC can be then plotted by calling

```
FRA::plotFRC(model = model)
```

whereas cell-to-cell heterogeneity pie-chart by calling

```
FRA::plotHeterogeneityPieCharts(model = model)
```

Supplementary Methods

1. Equivalency of Eq. 4 in the main paper and Eq. 11

More formally, the relationship between FRA and $C^*_{\min}$ can be derived in the following way. The fraction of cells stimulated with the dose k that can be decoded correctly is the fraction of the probability density, $P(y|x_k)$, with highest values among all doses $x_1,\ldots,x_i$,

$$v_{kk} = \int_{\mathcal{Y}_k} P(y|x_k)dy.$$

where $\mathcal{Y}_k$ is the set of responses y typical for the dose k

$\mathcal{Y}_k = \{y: P(y|x_k) > P(y|x_l) \text{ for } l \neq k \text{ from 1 to } i\}$.

Therefore, the sum of fractions of cells that can be decoded correctly among doses $x_1,\ldots,x_i$, gives the value of the FRC

$$r(x_i) = \int_{\mathcal{Y}} \max_{x_k \le x_i} P(y|x_k)dy = \sum_{k=1}^{i} \int_{\mathcal{Y}_k} P(y|x_k)dy = \sum_{k=1}^{i} v_{kk},$$

which demonstrates that definitions of FRC given by Eq. 4 and Eq. 11 are equivalent.

2. Cancer cell lines methods (corresponding to section 3.2 of Supplementary Note 3)

Human lung epithelial cell lines: A549 (CCL-185, ATCC) and CALU1 cells (HTB-54, ATCC) were cultured under standard conditions at 37°C in a humidified atmosphere of 5% CO_2/95% air in high glucose Dulbecco's Modified Eagle Medium (DMEM, ThermoFisher, #41965062) or in low glucose Roswell Park Memorial Institute 1640 (RPMI 1640, Corning, #10-040-CV) medium, respectively, supplemented with 10% fetal bovine serum (FBS, ThermoFisher, #10500064) and 1% penicillin-streptomycin solution (P/S, ThermoFisher, #15140122). For immunofluorescence A549 and CALU1 cells were plated in 96-well microplates with μClear® flat bottom (Greiner, #655090) at density 9×10^4 cells per well. After 24 h, cells were incubated with recombinant human interferon gamma (IFN-γ, ThermoFisher, #PHC4031) at concentrations 0-20 ng/mL for 30 min. Cells after stimulation were fixed with 3.7% paraformaldehyde (PFA, Sigma Aldrich, #P6148) for 10 min at room temperature, RT, then

permeabilized with 90% ice-cold methanol (Sigma, #322415), for 30 min at -20°C, blocked with 5% bovine serum albumin (BSA, Merck, #821006) and 0.3% Triton X-100 (Sigma Aldrich, #T9284) for 1 h at RT, and incubated with primary antibody - phospho-STAT1 (Tyr701) (pSTAT1, Cell Signaling, #9167) diluted 1:100 in 1% BSA with 0.3% Triton X-100 overnight at 4°C. Next day, cells were incubated with an appropriate secondary antibody - Alexa Fluor 488 (Life Technologies, #A-21206) diluted 1:500 in 1% BSA with 0.3% Triton X-100 for 1.5 h at RT and stained with 2 μg/mL 4',6-diamidino-2-phenylindole (DAPI, Sigma Aldrich, #D9542) for 10 min at RT. The fluorescence signal was acquired and quantified using an automated confocal microscope (Pathway 435, BD) and analyzed with Cell Profiler v2.1.1 and ImageJ v1.48.

Supplementary References

1. Van Erven, T. & Harremos, P. Rényi divergence and Kullback-Leibler divergence. *IEEE Transactions on Information Theory* 60, 3797–3820 (2014).
2. Shtarkov, Y. M. Universal sequential coding of single messages. *Problemy Peredachi Informatsii* 23, 3–17 (1987).
3. Rényi, A. On Measures of Entropy and Information. in 1, 547–561 (University of California Press, Berkeley, Calif., 1961).
4. Verdú, S. α-mutual information. in *2015 Information Theory and Applications Workshop (ITA)* 1–6 (IEEE, 2015). doi:10.1109/ITA.2015.7308959
5. Cover, T. M. & Thomas, J. A. *Elements Of Information Theory*. 792 (John Wiley & Sons, 2012).
6. Yeung, R. W. *Information Theory and Network Coding*. (Springer Science & Business Media, 2008).
7. Jetka, T., Nienałtowski, K., Filippi, S., Stumpf, M. P. H. & Komorowski, M. An information-theoretic framework for deciphering pleiotropic and noisy biochemical signaling. *Nat. Commun.* 9, 4591 (2018).
8. Cheong, R., Rhee, A., Wang, C. J., Nemenman, I. & Levchenko, A. Information transduction capacity of noisy biochemical signaling networks. *Science* 334, 354–358 (2011).
9. Suderman, R., Bachman, J. A., Smith, A., Sorger, P. K. & Deeds, E. J. Fundamental trade-offs between information flow in single cells and cellular populations. *Proc. Natl. Acad. Sci. USA* 114, 5755–5760 (2017).
10. Selimkhanov, J. *et al.* Accurate information transmission through dynamic biochemical signaling networks. *Science* 346, 1370–1373 (2014).
11. Jetka, T., Nienałtowski, K., Winarski, T., Błoński, S. & Komorowski, M. Information-theoretic analysis of multivariate single-cell signaling responses. *PLoS Comput. Biol.* 15, e1007132 (2019).
12. Keshelava, A. *et al.* High capacity in G protein-coupled receptor signaling. *Nat. Commun.* 9, 876 (2018).

Supplementary Tables

Target	Phenotypic	Clone	Metal label	Company	Catalogue #	Dilution	Location
CD14	Yes	TUK4	Qdot655	Thermo Fisher	Q10056	1:200	surface (pre-fixation)
CD45	Yes	HI30	89Y	Fluidigm	3089003B	1:100	surface
CD11c	Yes	Bu15	141Pr	Biolegend	337221	1:100	surface
CD11b	Yes	ICRF44	142Nd	Biolegend	301337	1:100	surface
CD45RA	Yes	HI100	143Nd	Biolegend	304143	1:50	surface
HLA-DR	Yes	L243	144Nd	Biolegend	307651	1:100	surface
CD4	Yes	RPA-T4	145Nd	Biolegend	300541	1:100	surface
CD19	Yes	HIB19	146Nd	Biolegend	302247	1:100	surface
CD20	Yes	2H7	147Sm	Biolegend	302343	1:100	surface
CCR6	Yes	G034E3	148Nd	Biolegend	353427	1:100	surface (pre-fixation)
CD56	Yes	NCAM16.2	149Sm	Fluidigm	3149021B	1:50	surface (pre-fixation)
p-STAT5	No	47	150Nd	Fluidigm	3150005A	1:50	intracellular
CD45RO	Yes	UCHL1	151Eu	Biolegend	304239	1:50	surface (pre-fixation)
CD27	Yes	O323	152Sm	Biolegend	302839	1:100	surface (pre-fixation)
p-STAT1	No	4a	153Eu	Fluidigm	3153005A	1:50	intracellular
CD1c	Yes	L161	154Sm	Biolegend	331502	1:50	surface
CD123	Yes	6H6	155Gd	Biolegend	306027	1:50	surface
p-p38	No	D3F9	156Gd	Fluidigm	3156002A	1:50	intracellular
p-STAT3	No	4/P-Stat3	158Gd	Fluidigm	3158005A	1:50	intracellular
p-MAPKAPK2	No	27B7	159Tb	Fluidigm	3159010A	1:50	intracellular
CD3	Yes	UCHT1	160Gd	Biolegend	300443	1:100	surface
DNGR1	Yes	8F9	161Dy	Fluidigm	3161018B	1:50	surface
IFNAR2	No	polyclonal	162Dy	Abcam	ab56070	1:50	surface
STAT1	No	246523	163Dy	Bio-Techne	MAB1490	1:50	intracellular
IFNAR1	No	EP899Y	164Dy	Abcam	ab213331	1:50	surface
CD161	Yes	HP-3G10	165Ho	Biolegend	339919	1:50	surface
p-NFkBp65	No	K10x	166Er	Fluidigm	3166006A	1:50	intracellular
CCR7	Yes	G043H7	167Er	Fluidigm	3167009A	1:50	surface (pre-fixation)
p-STAT6	No	18/P-Stat6	168Er	Fluidigm	3168012A	1:50	intracellular
CD24	Yes	ML5	169Tm	Fluidigm	3169004B	1:50	surface
CD141	Yes	M80	170Er	Biolegend	344102	1:50	surface
p-ERK1/2	No	D13.14.4E	171Yb	Fluidigm	3171010A	1:50	intracellular
CD38	Yes	HIT2	172Yb	Fluidigm	3172007B	1:50	surface
STAT3	No	124H6	173Yb	Fluidigm	3173003A	1:100	intracellular
p-STAT4	No	38/p-Stat4	174Yb	Fluidigm	3174005A	1:50	intracellular
CCR4	Yes	L291H4	175Lu	Fluidigm	3175035A	1:50	surface (pre-fixation)
CXCR3	Yes	G025H7	176Yb	Biolegend	353733	1:50	surface (pre-fixation)
CD8	Yes	RPA-T8	198Pt	Biolegend	301053	1:100	surface
CD16	Yes	3G8	209Bi	Fluidigm	3209002B	1:100	surface

Supplementary Table 1: Antibodies used for mass cytometry experiments.

Data type	Interpretation term	Increase in FRC $r(x_i)$- $r(x_{i-1})$	Value of FRC $r(x_i)$	Value of FRC for the highest dose, $2^{C^{*}min}$	Pie-chart diagonal elements	Pie-chart off - diagonal elements (bands around FRC)
**Response distributions of populations of single-cells**	**Fractions**	Fraction of cells stimulated with x_i with responses distinct from all lower doses (more likely for x_i than for any lower dose).	Sum of fractions of cells stimulated with doses not greater than x_i with responses typical (most likely) to the encountered dose.	Overall sum of fraction of cells with responses typical for the encountered dose.	Fraction of cells stimulated with a given dose with responses typical (most likely) for that dose.	Fraction of cells stimulated with a given dose with responses typical (most frequent) for any of the other doses.
	**Probability**	Probability of a cell in the population stimulated with x to generate a response that is distinct from all lower doses (more likely for x_i than for any of the lower doses)	Number of distinct response distributions for doses not greater than x_i	Overall number of distinct response distributions.	Probability of a cell in the population stimulated with a given does to generate a response that is typical (most frequent) for the encountered dose.	Probability of a cell in the population stimulated with a given does to generate a response that is typical (most frequent) for a given dose other than the encountered one.
	**Decoding by an external observer** using maximum likelihood principle	Probability of correctly discriminating the dose x_i from lower doses.	Sum of probabilities of correct decoding of the doses not greater than x_i,. This probability divided by i gives the probability of correctly decoding doses not greater than x_i.	The overall sum of probabilities of correct decoding. This probability divided by the number of doses, m, gives the overall probability of correct decoding.	Probability of correctly decoding a given dose.	Probability of confusing one dose with another dose.

Supplementary Table 2: The summary of FRA interpretation: FRA can be interpreted in terms of fractions of cells, probabilities of responses, as well as information-theoretic decoding framework.

Supplementary Figures

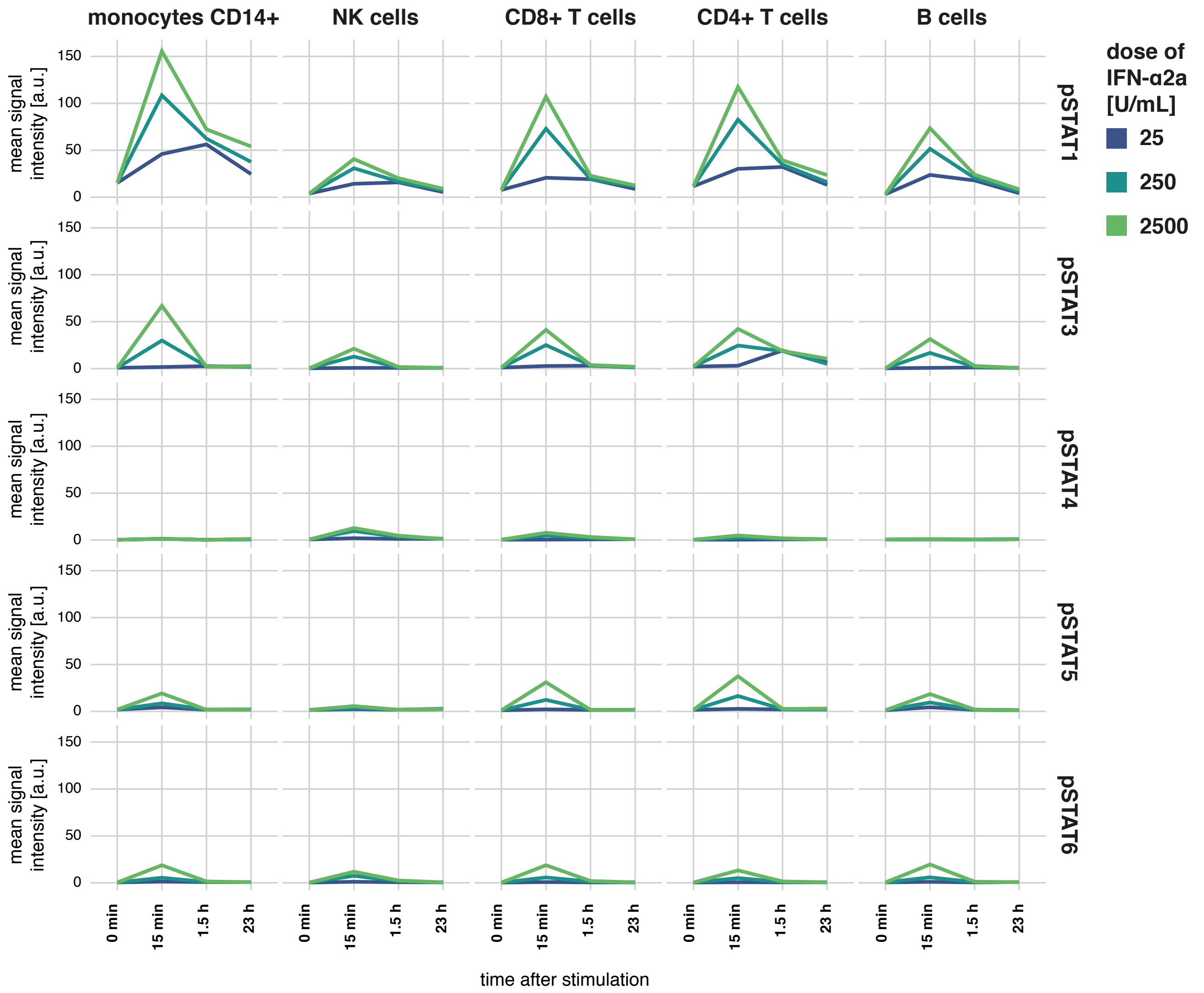

Supplementary Figure 1. Time-course of responses to IFN-α2a in PBMCs. Population mean response (y-axis) of each cell type (columns) is shown in terms of whole cell levels of different pSTATs (rows), as measured with mass cytometry for different time points (x-axis) after stimulation with the indicted dose (color). Technical details: mean responses were calculated based on at least 2500 cells per condition.

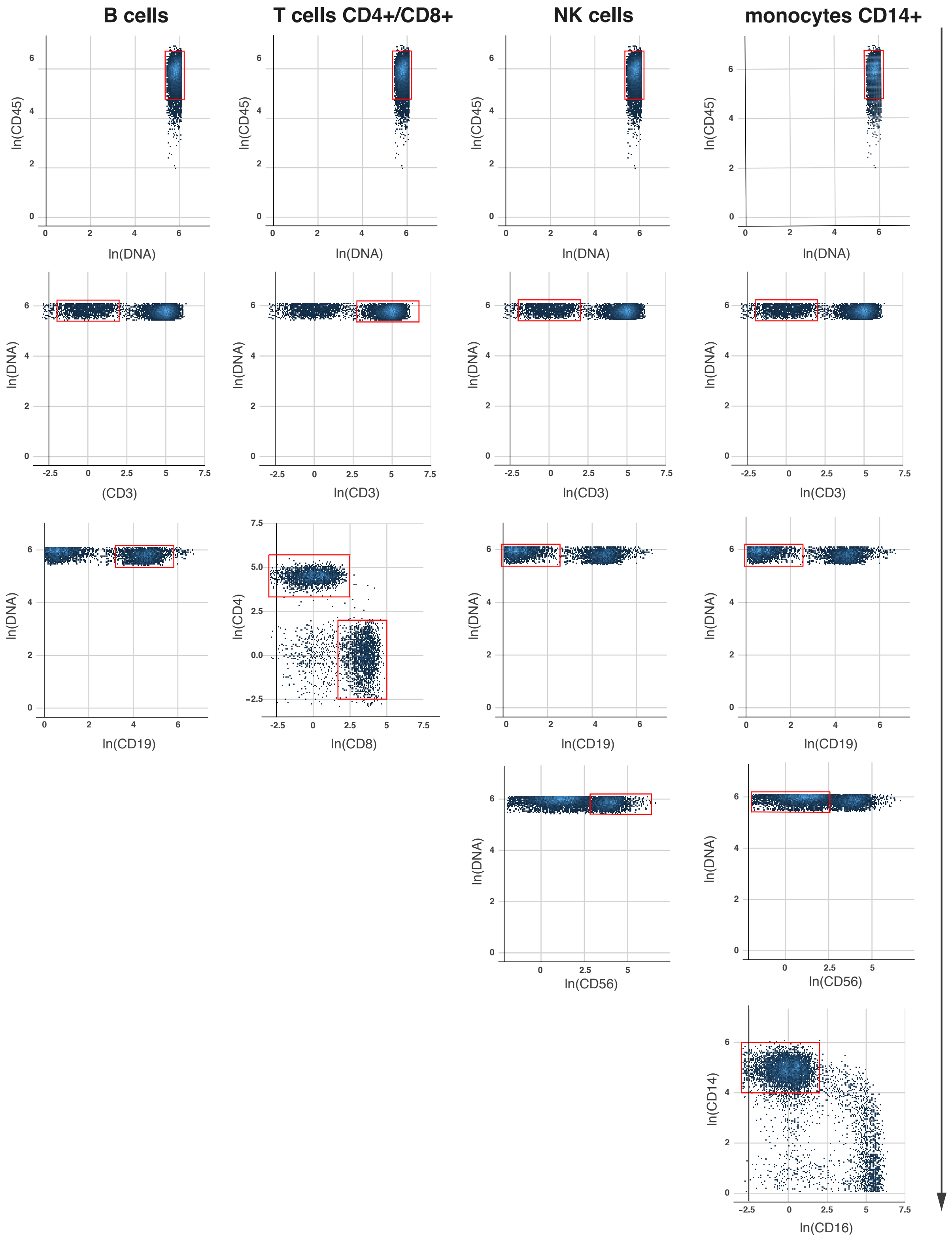

Supplementary Figure 2. Representative manual gating strategy of different immune cell subpopulations. Lineage markers CD3 and CD19 were firstly used to define CD3+ T cells and CD19+ B cells from the live, single, CD45+ cells. Within the CD3 T cell population, CD4+ and CD8+ T cells were identified. CD3- CD19- cells were assessed for expression of the NK cell marker CD56. CD3-CD19-CD56- cells were assessed for expression of the monocyte marker CD14, and further gated based on their expression of CD16. B cells, T cells CD4+, T cells CD8+, NK cells and monocytes CD14+ gated with the presented strategy were used for the plotting data presented on Fig. 1, Fig 3, Sup. Fig. 1, 3, 4, 7 and 8.

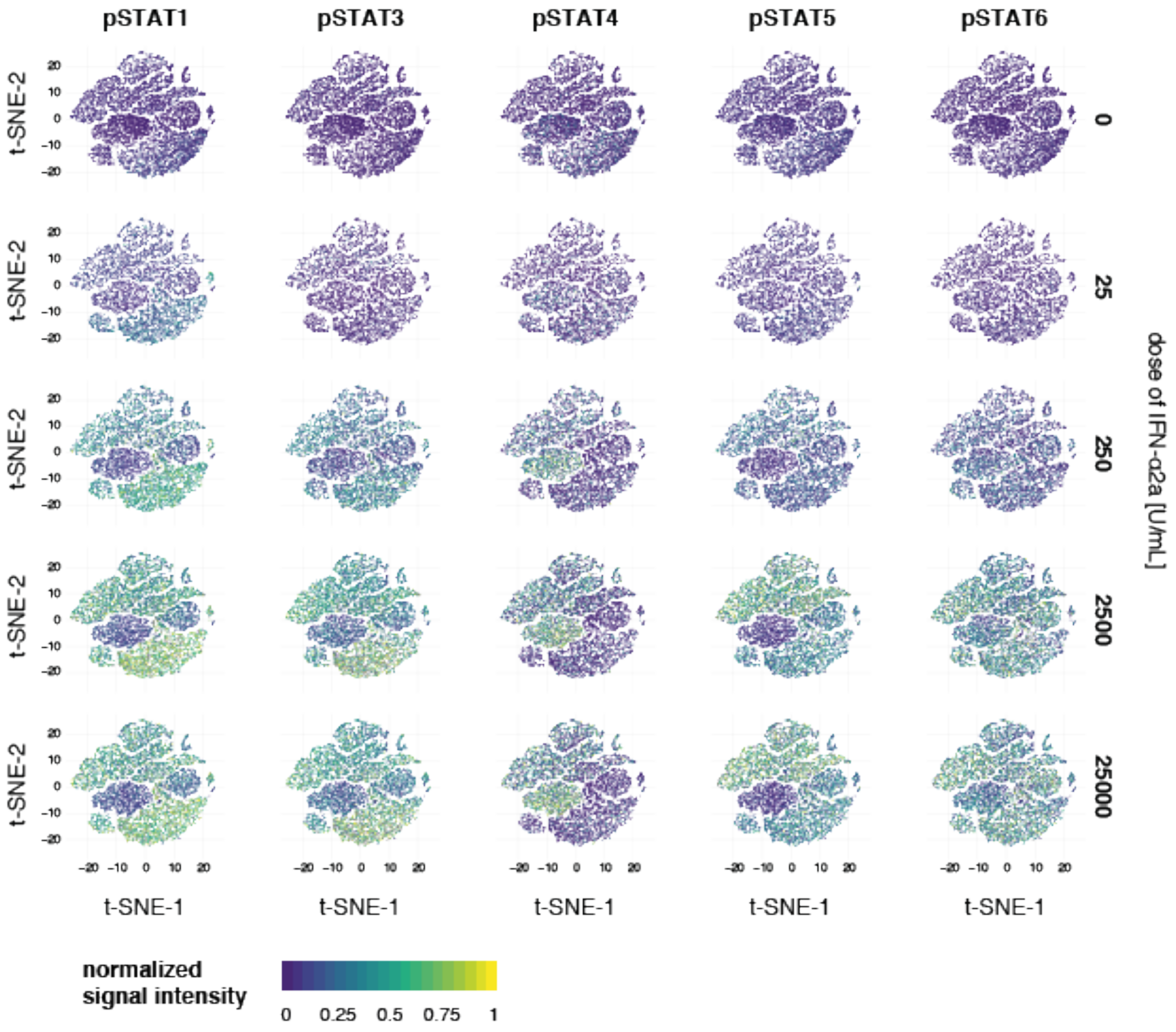

Supplementary Figure 3. **Dose-responses to IFN-α2a in PBMCs presented as t-SNE plots.**
Figure corresponds to Fig. 1b, where responses to selected doses are presented. Here all doses are shown.

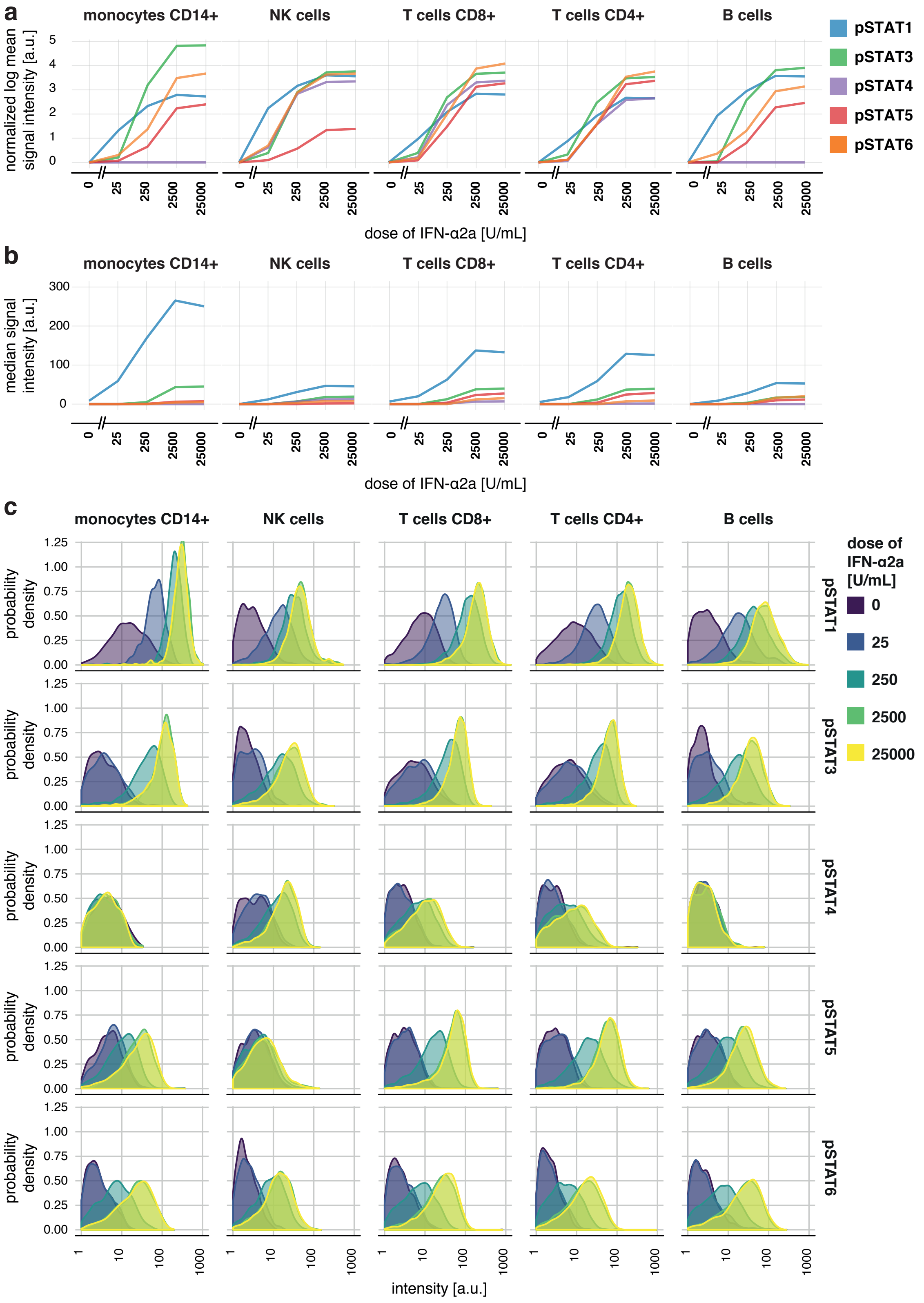

Supplementary Figure 4. **Dose-responses to IFN-α2a.**

a Mean of log pSTATs levels. The panel corresponds to Fig. 1c, where mean responses are shown.

b Medians of pSTATs levels. The panel also corresponds to Fig. 1c.

c Population distributions of pSTATs levels. The panel corresponds to Fig. 1d, where only distributions of pSTAT1 and pSTAT5 are shown. Here all pSTATs are shown.

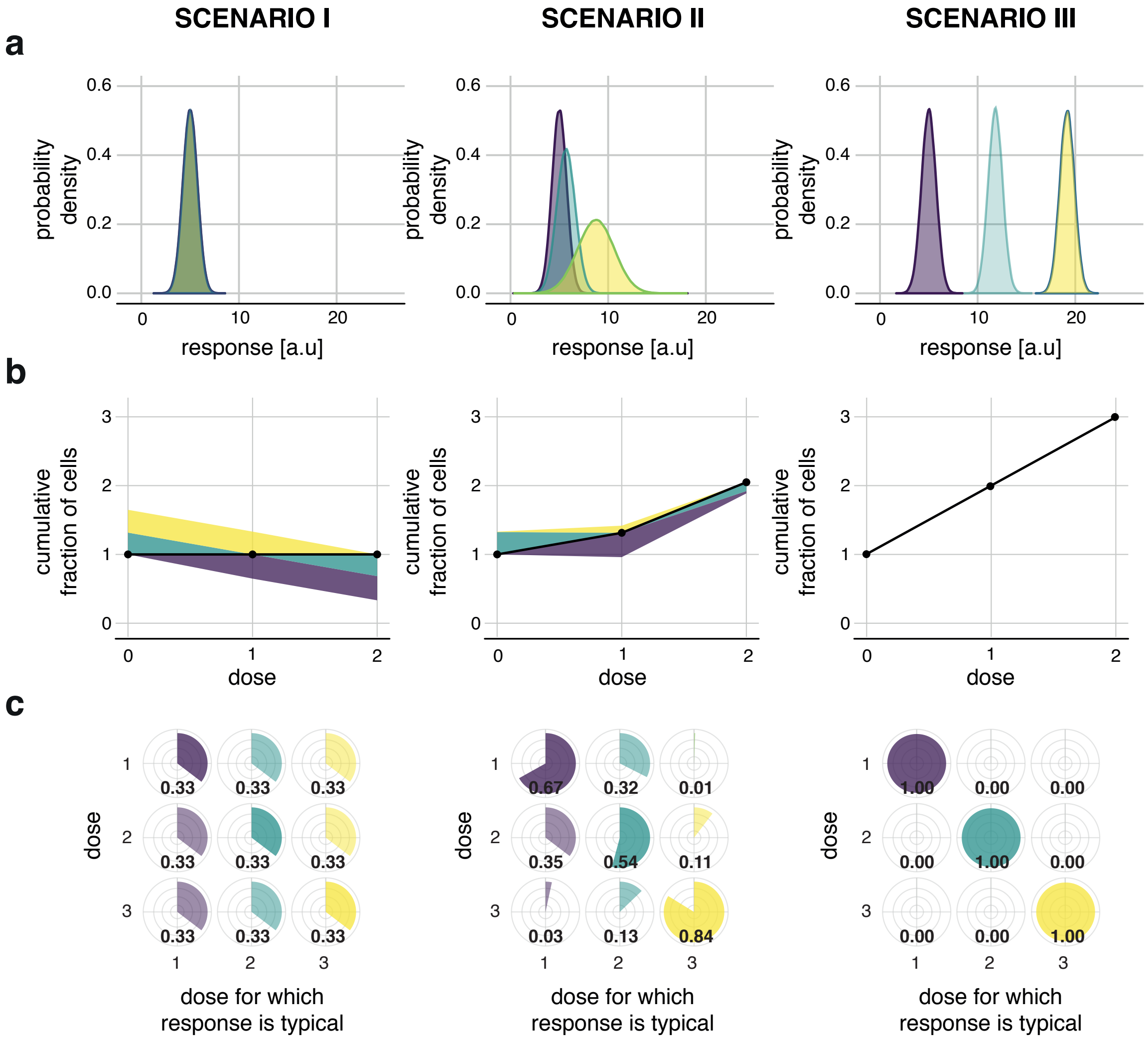

Supplementary Figure 5. **FRC counts the number of distinct response distributions** (Figure corresponds to Fig. 2).
a Distributions of responses in three hypothetical scenarios. Scenario I: completely overlapping responses; Scenario II: the same as in Fig. 2, i.e., partly overlapping responses; Scenario III: completely distinct responses.
b FRA in the three scenarios (columns).
c Pie-charts representing cell-to-cell heterogeneity structure in the three scenarios (columns).

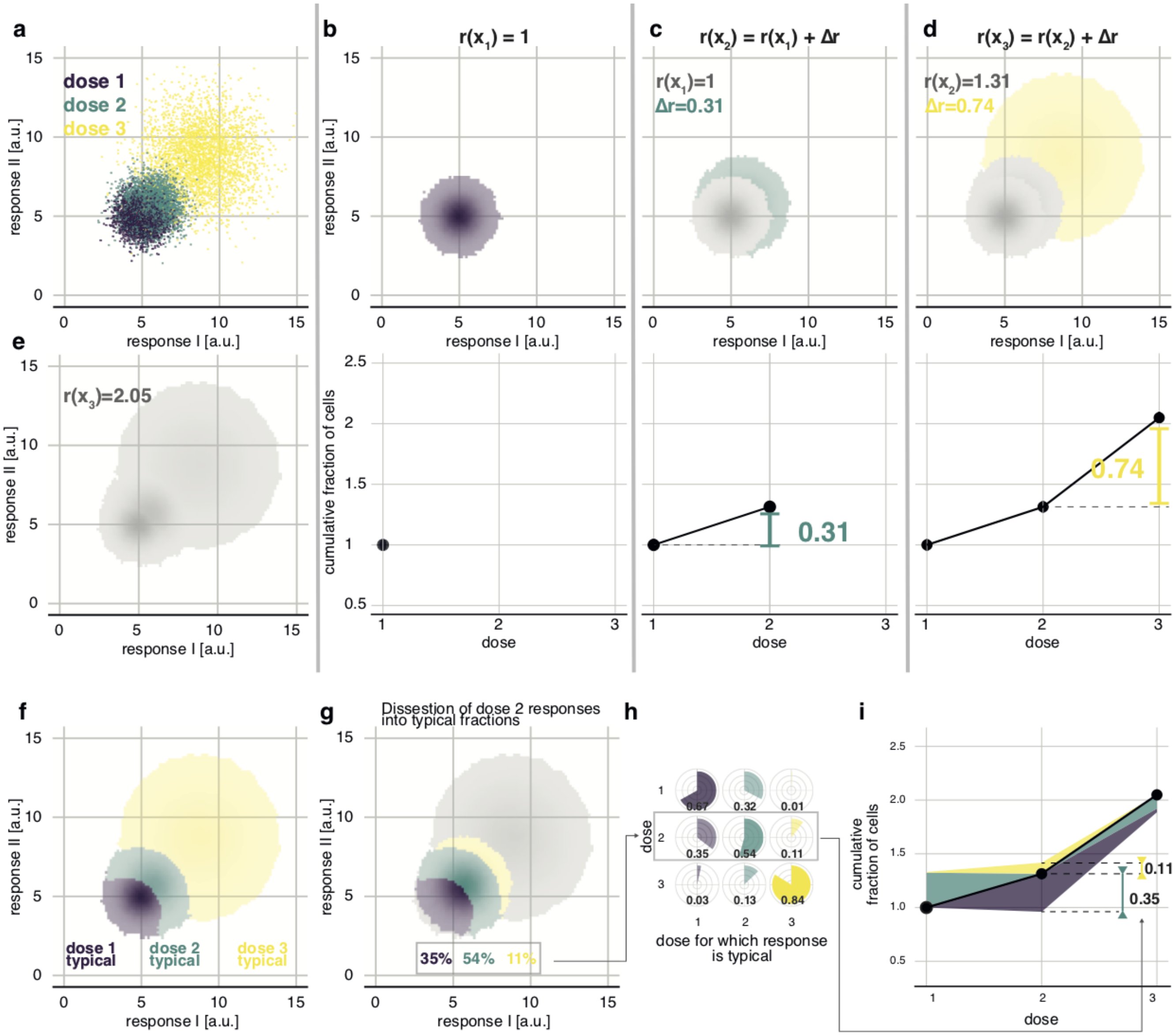

Supplementary Figure 6. **Fractional response analysis illustrated for two dimensional data (analogous to one dimensional Fig. 2).**

a Hypothetical response two dimensional distributions to three different doses encoded by colors. Covariates are referred to as response I and response II

b-d Quantification of the fraction of cells that exhibit different responses due to dose increase, Δr, and constriction of FRC, for responses presented in **a**.

e Quantification of the number of distinct distributions induced by the three considered doses.

f Dose-typical responses for the response distributions of **a**.

g Dissection of the responses to dose 2 into responses typical to any of the three doses.

h The fractions of cells stimulated with one dose (rows) with responses typical to any of the doses (columns).

i The FRC together with the bands representing cell-to-cell heterogeneity as quantified in **h**.

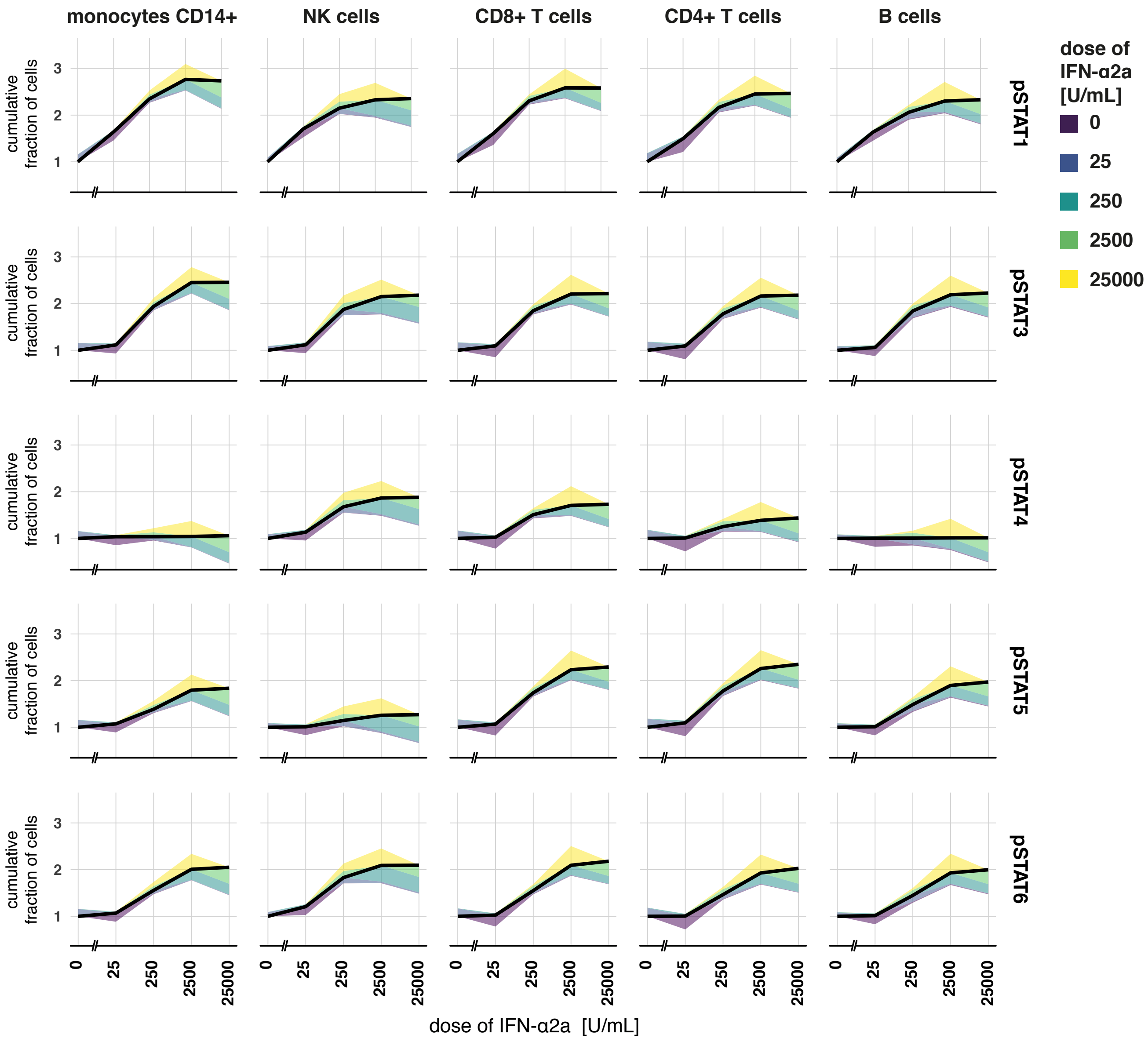

Supplementary Figure 7. **FRA of IFN-α2a responses for individual STAT proteins.**
Figure corresponds to Fig. 3a, where FRA were performed assuming that all pSTATs jointly constitute cell's response. Here, each panel presents FRA for an individual pSTAT (rows) for different cell types (columns). Data used to plot each panel is shown in the corresponding panel of Supplementary Figure 3.

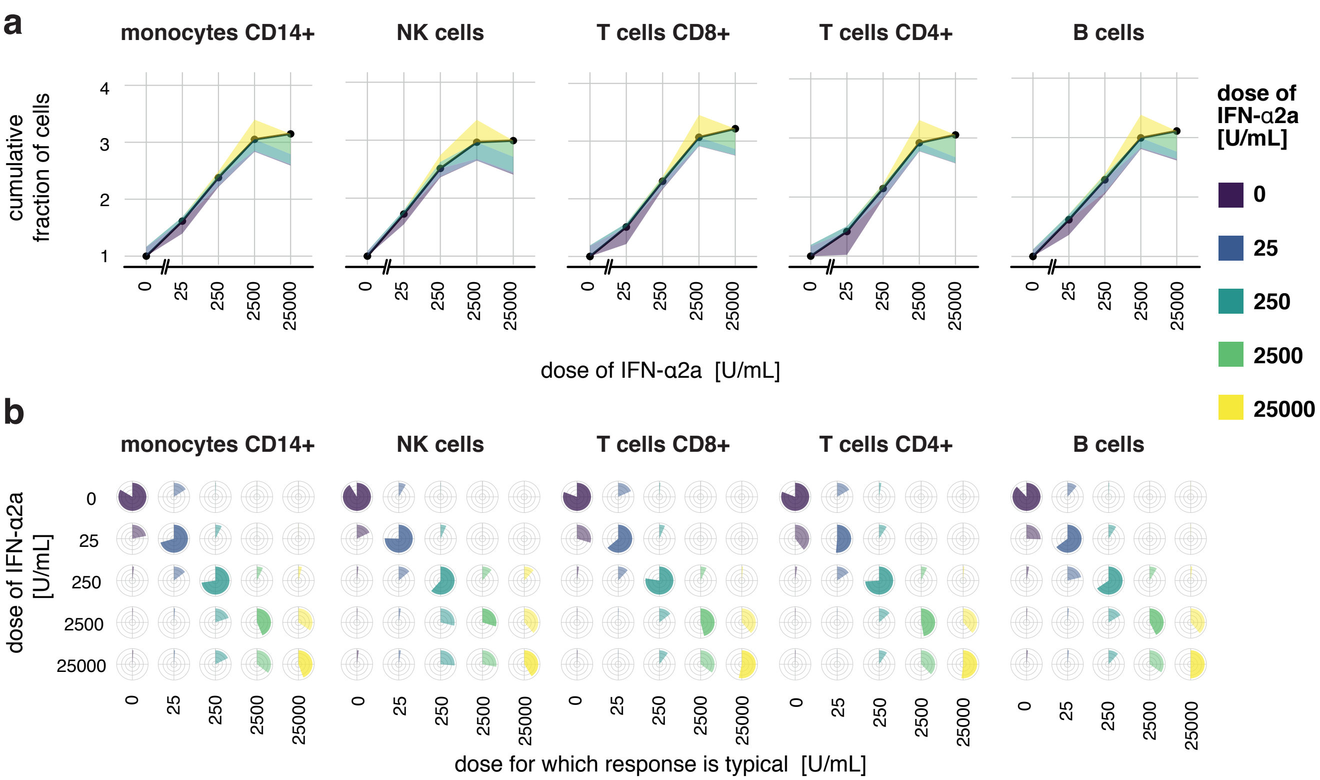

Supplementary Figure 8. An independent biological replicate of Fig. 3.
a FRA of IFN-α2a responses.
b Piecharts of cell-to-cell heterogeneity structure.Technical details: at least 2500 cells were measured per condition.

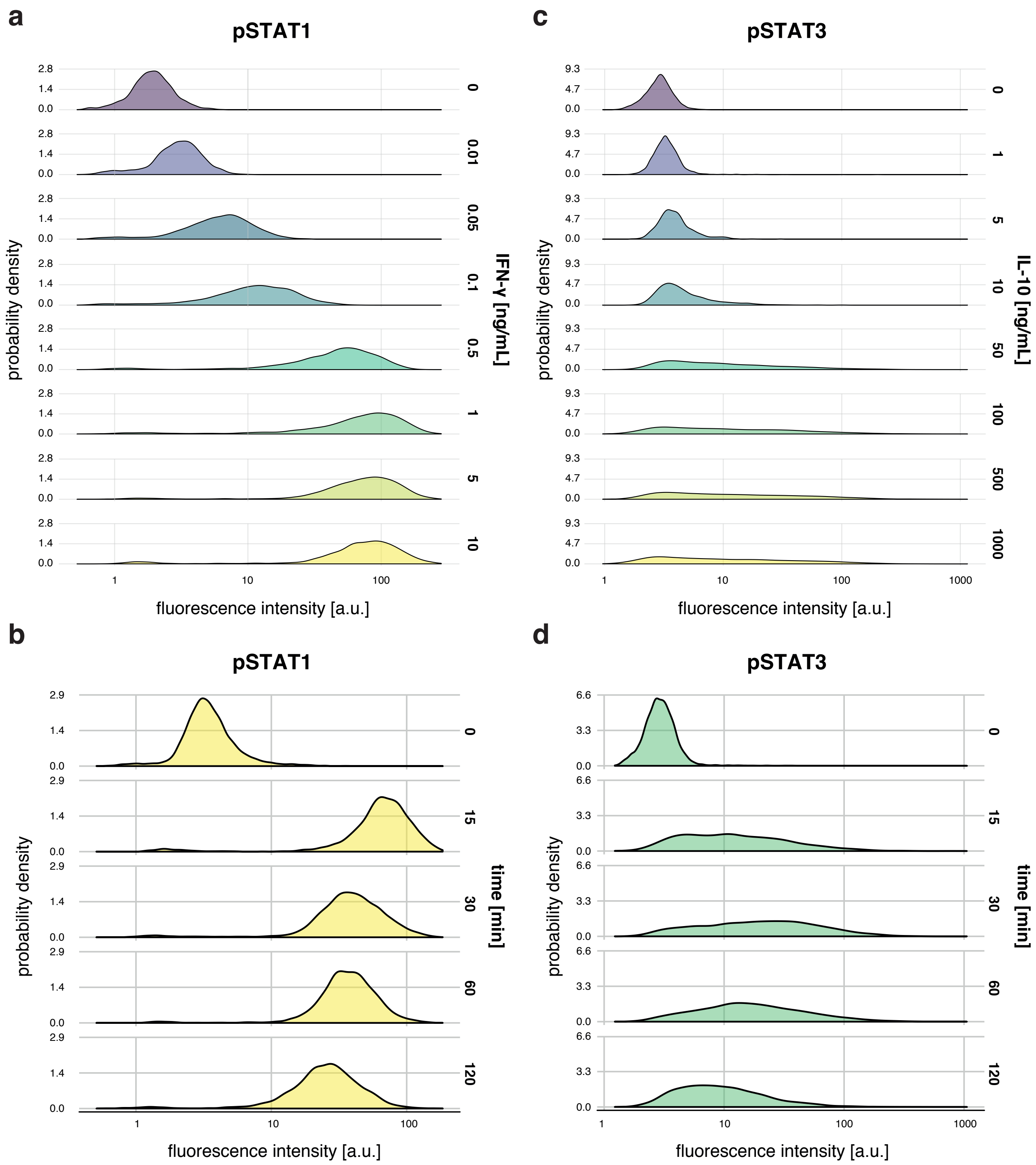

Supplementary Figure 9. IFN-γ, IL-10 response distributions (Figure corresponds to Fig. 4a,b).
a Response distributions to IFN-γ as in Fig. 4a were response distributions to selected doses are shown. Here all doses are shown.
b Same as in **a** but for IL-10, corresponds to Fig. 4b.
c Distributions of responses for different times after stimulation with 10 ng/mL of IFN-γ.
d Distributions of responses for different times after stimulation with 100 ng/mL of IL-10.

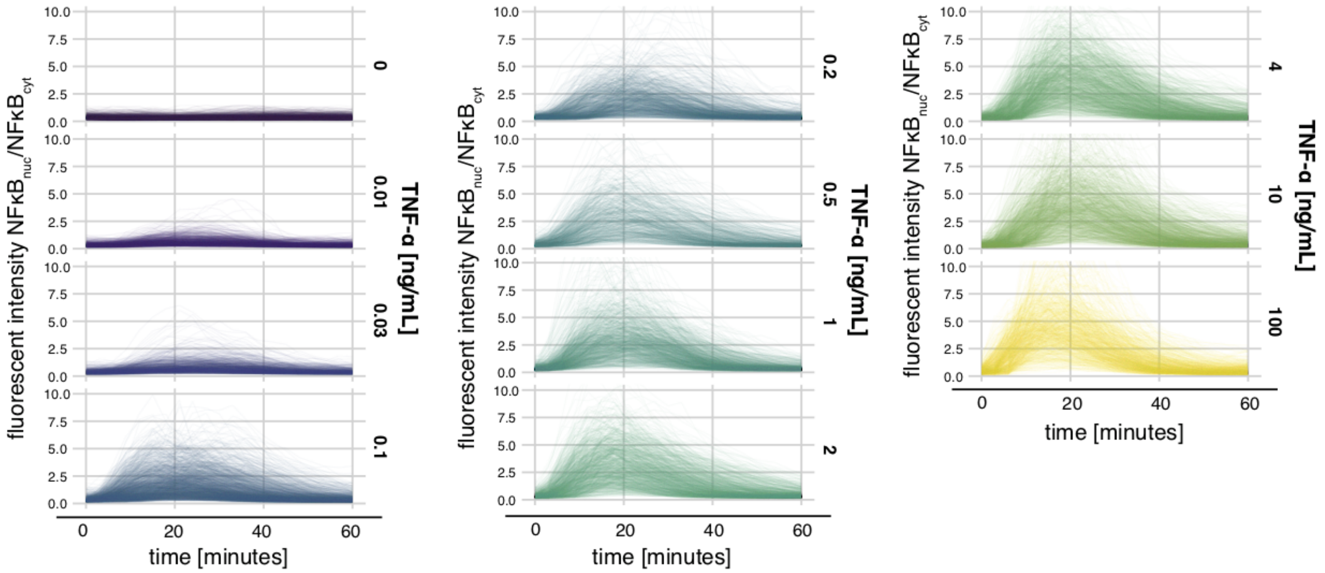

Supplementary Figure 10. Temporally resolved responses to TNF-α.
Figure corresponds to Fig. 4c, where responses to selected doses are presented. Here all doses are shown.

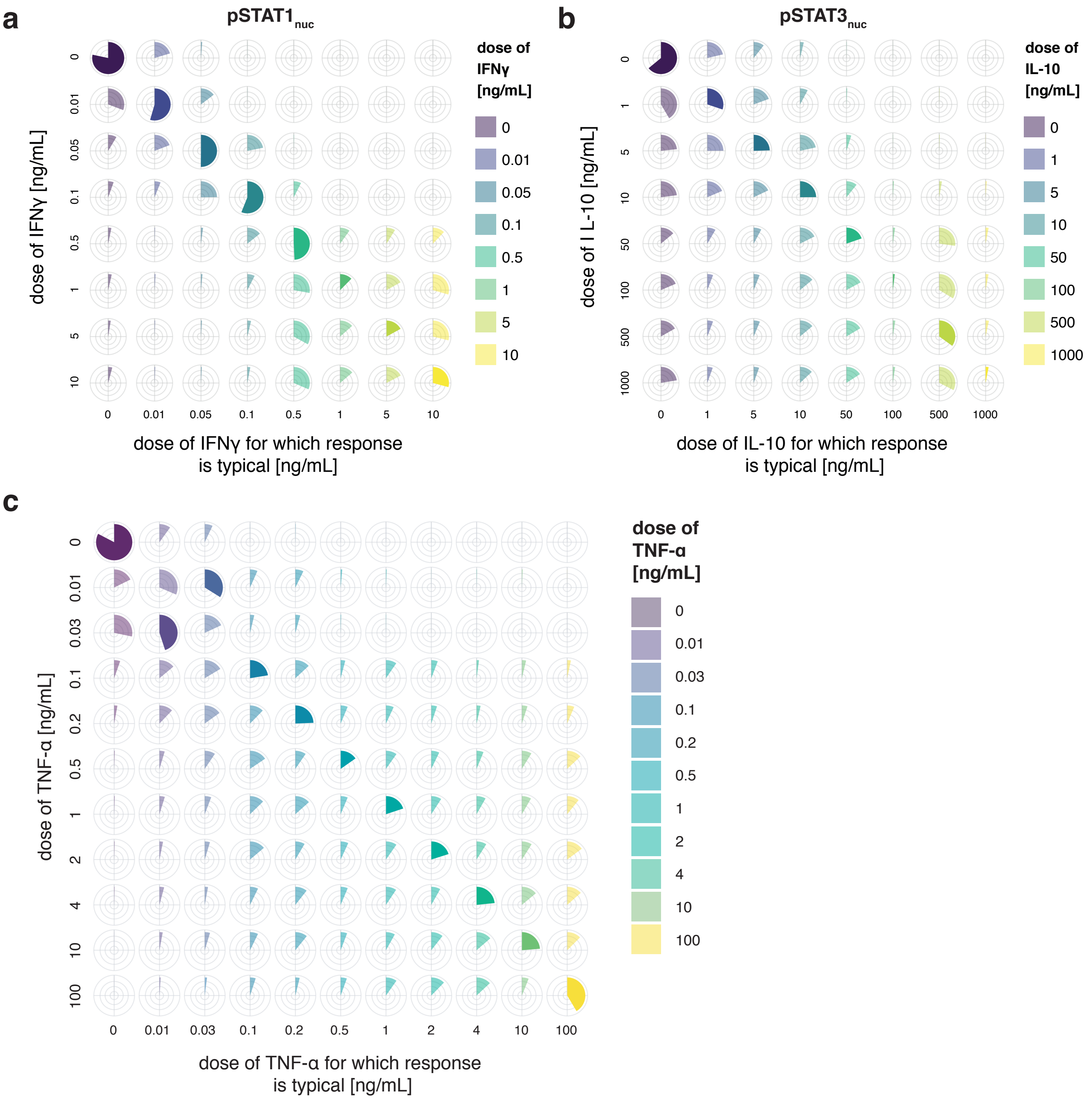

Supplementary Figure 11. **Pie-charts of the cell-to-cell heterogeneity structure used to plot color bands in Fig. 4d-f.**
a Cell-to-cell heterogeneity structure of IFN-γ responses, corresponds to Fig. 4d.
b Cell-to-cell heterogeneity structure of IL-10 responses, corresponds to Fig. 4e.
c Cell-to-cell heterogeneity structure of NF-κB responses, corresponds to Fig. 4f.

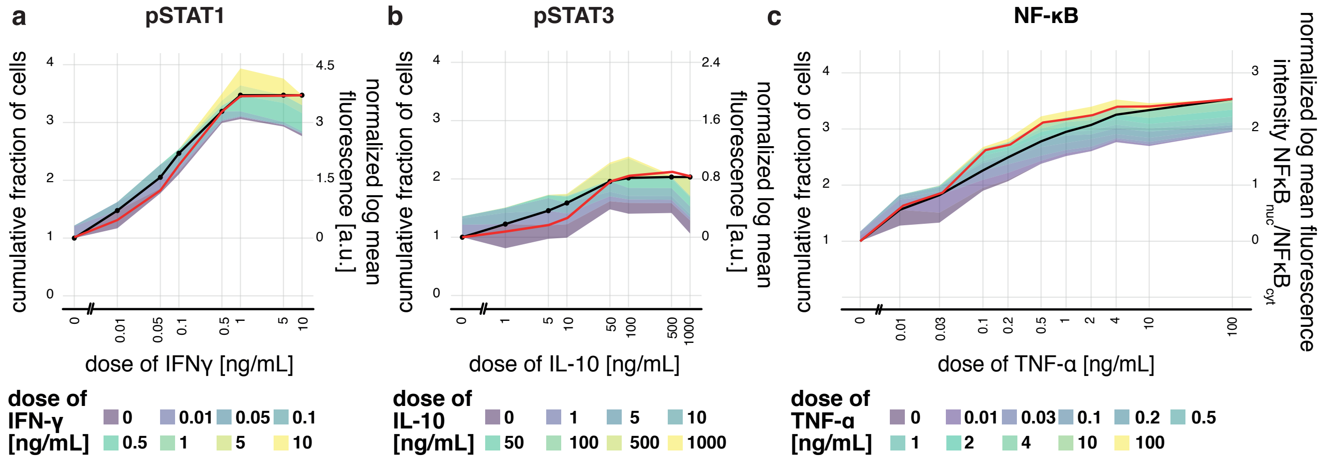

Supplementary Figure 12. Comparison of FRA for IFN-γ, IL-10, and TNF-α responses with mean response of log-data, analogously to Fig.4d–f, where FRC and mean responses are shown.

a FRA of IFN-γ responses. For comparison, the red line presents the mean of log response with y-axis on the right. **b** Same as in a but IL-10 response.

c Same as in a but TNF-α response for a single time-point (18 min.) after stimulation.

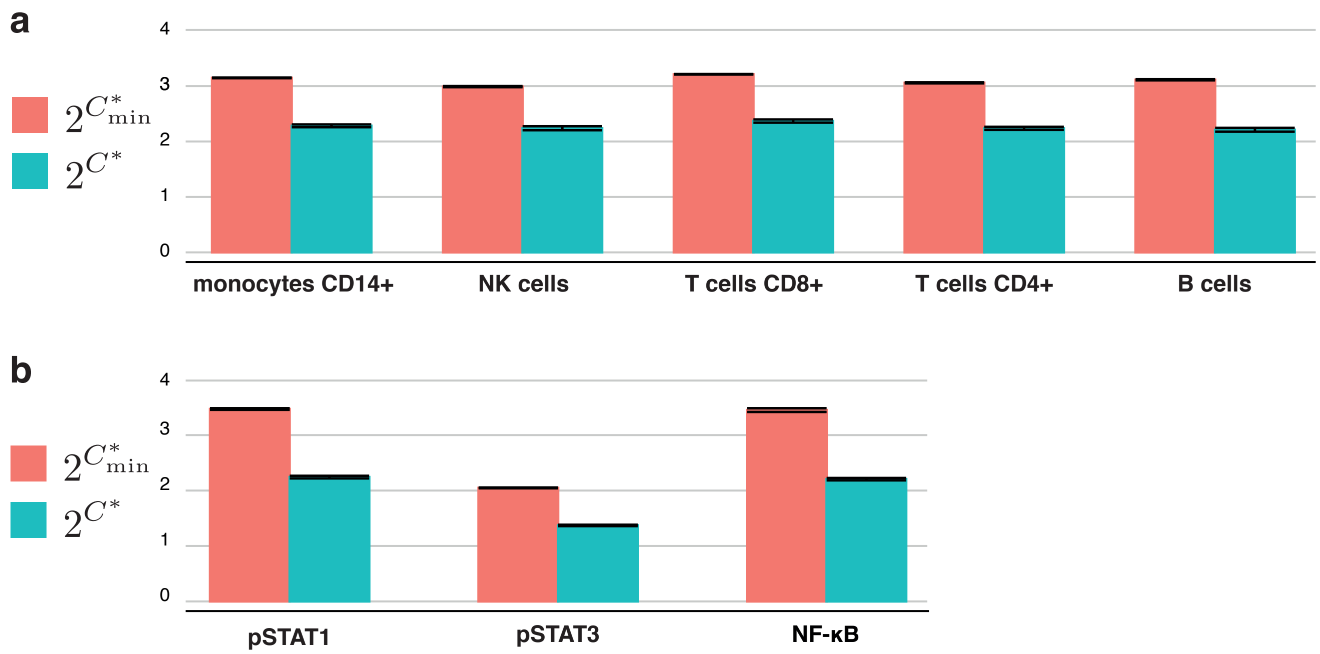

Supplementary Figure 13. Comparison of Rényi min-capacity and Shannon capacity for the data presented in the main paper.

a Rényi min-capacity (red) and Shannon capacity (green) presented in terms of power of 2 calculated for IFN-α2a responses in PBMCs analyzed in Fig. 1.

b The same as in **a** but calculated for IFN-γ, IL-10, TNF-α response analyzed in Fig. 4. Technical details: capacities were calculated with bootstrap involving 128 resamplings. Bars present mean values +/- standard deviation.

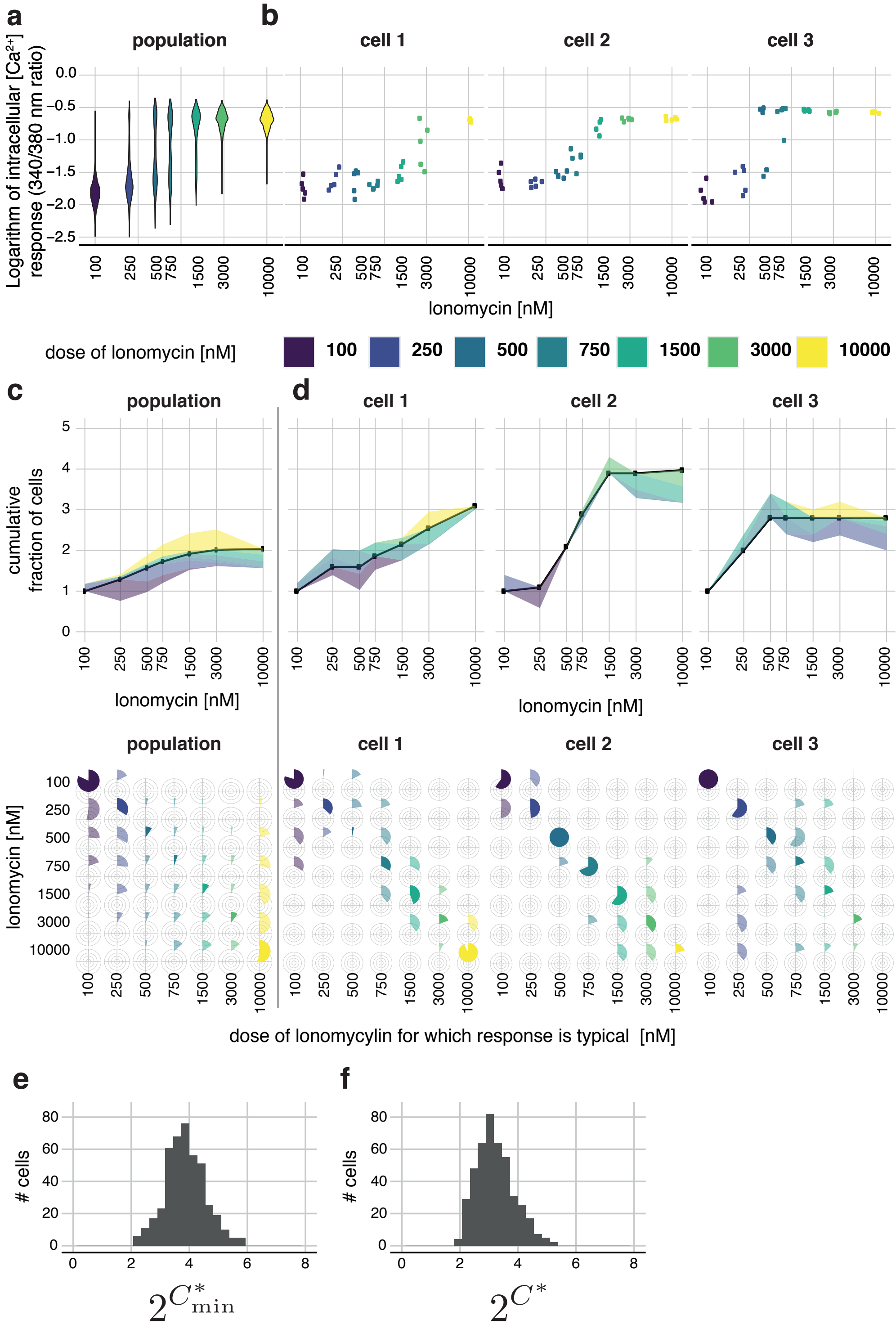

Supplementary Figure 14. Comparison of Rényi min-capacity and Shannon capacity for GPCR signaling published in ***Keshelava et al. Nat Commun 2018***[12]**.**

a Violin plot representing population response distributions to different doses of Ionomycin. At least 433 cells were measured per condition, whereas each cell was measured 35 times[11].

b Responses of three representative cells to different doses of Ionomycin. Points at each panel correspond to measurements performed in the same cell.

c FRA of the population responses shown in **a**.

d FRA of the responses of three individual cells shown in **b**.

e Variability of Shannon capacity in the cellular population. Shannon capacity, represented here as power of 2, was calculated for each cell and presented as histogram.

f Same as in **e** but for Rényi min-capacity.

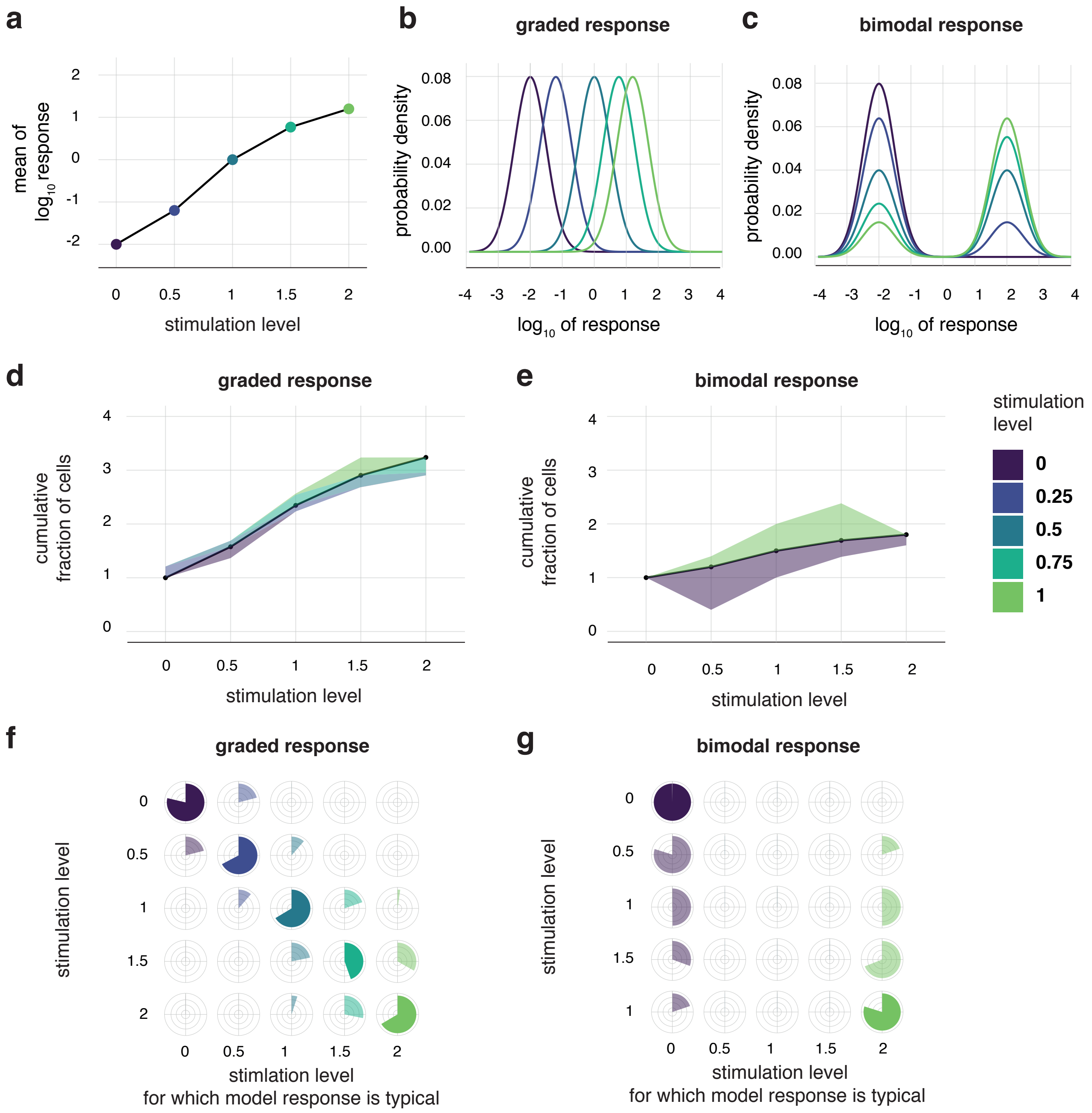

Supplementary Figure 15. FRA of binary vs graded response for the in silico model.
a Mean of log responses in the binary and graded *in silico* models.
b Distribution of log responses in the graded models.
c Distribution of log responses in the binary models.
d FRA of distribution of log responses in the graded models shown in **b**.
e FRA of distribution of log responses in the binary models shown in **c**.
f Pie-charts corresponding to **d**, representing cell-to-cell heterogeneity structure in the graded model.
g Pie-charts corresponding to **d**, representing cell-to-cell heterogeneity structure in the binary model.

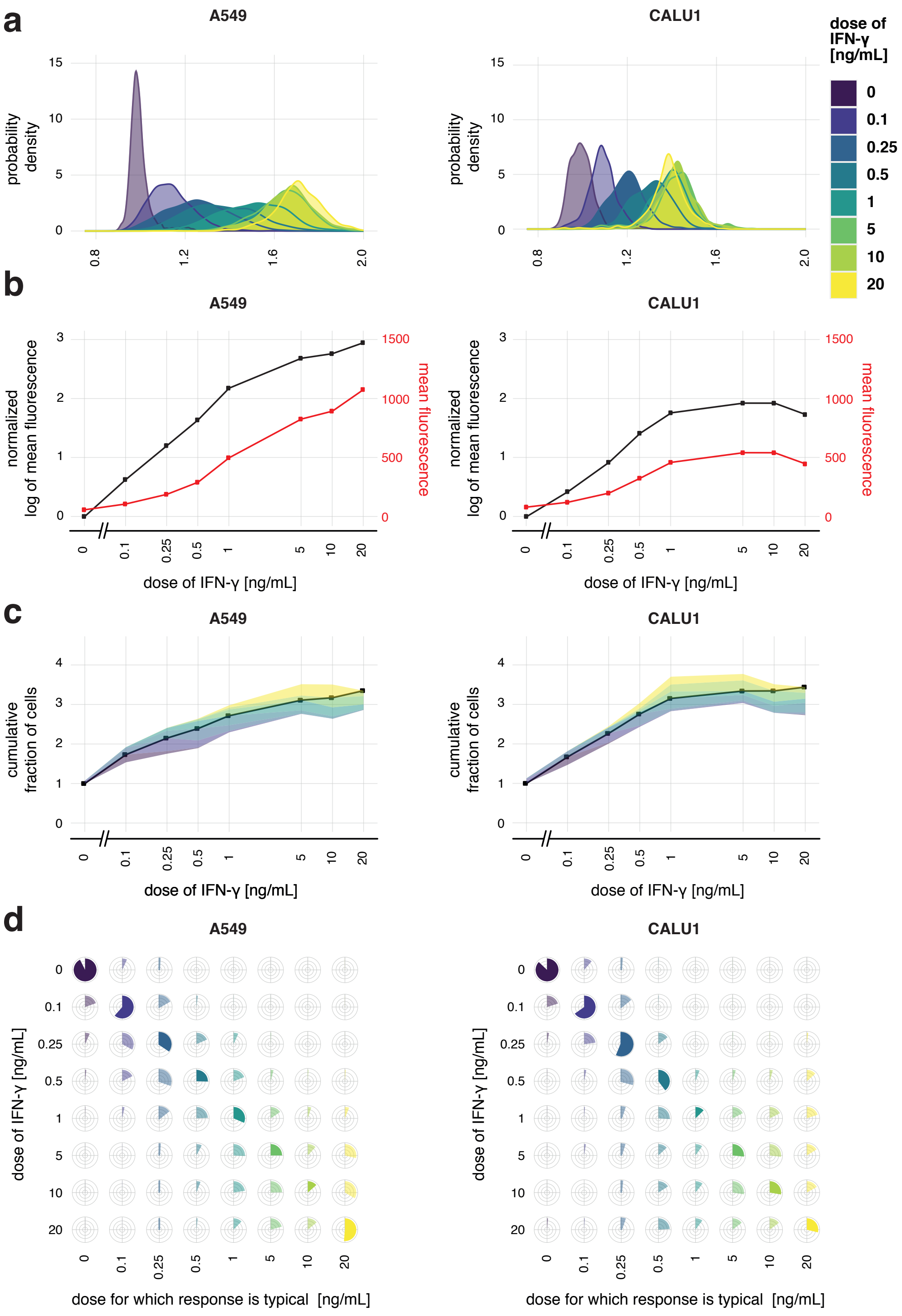

Supplementary Figure 16. Sensitivity of IFN-γ responses in lung cancer cell lines.
a Distributions of responses 30 min after stimulation with different doses of IFN-γ in terms of nuclear pSTAT1 as measured with confocal microscopy imaging and immunostaining in the A549 (left panel) and CALU1 (right panel) cell lines. Responses are expressed as mean fluorescence of nuclear pixels.
b Mean pSTATs levels (red line) and means of logs of pSTATs levels (black line) in the two cell lines as a function of dose.
c FRA of responses shown in **a**.
d Pie-charts, corresponding to **c**, representing cell-to-cell heterogeneity structure of population responses shown in **a**.
Technical details: at least 811 A549 and 351 CALU1 cells were measured per conditions. Experiments were performed in two biological replicates, each containing at two technical replicates. Shown is the sum of two technical replicates of one representative biological replicate.

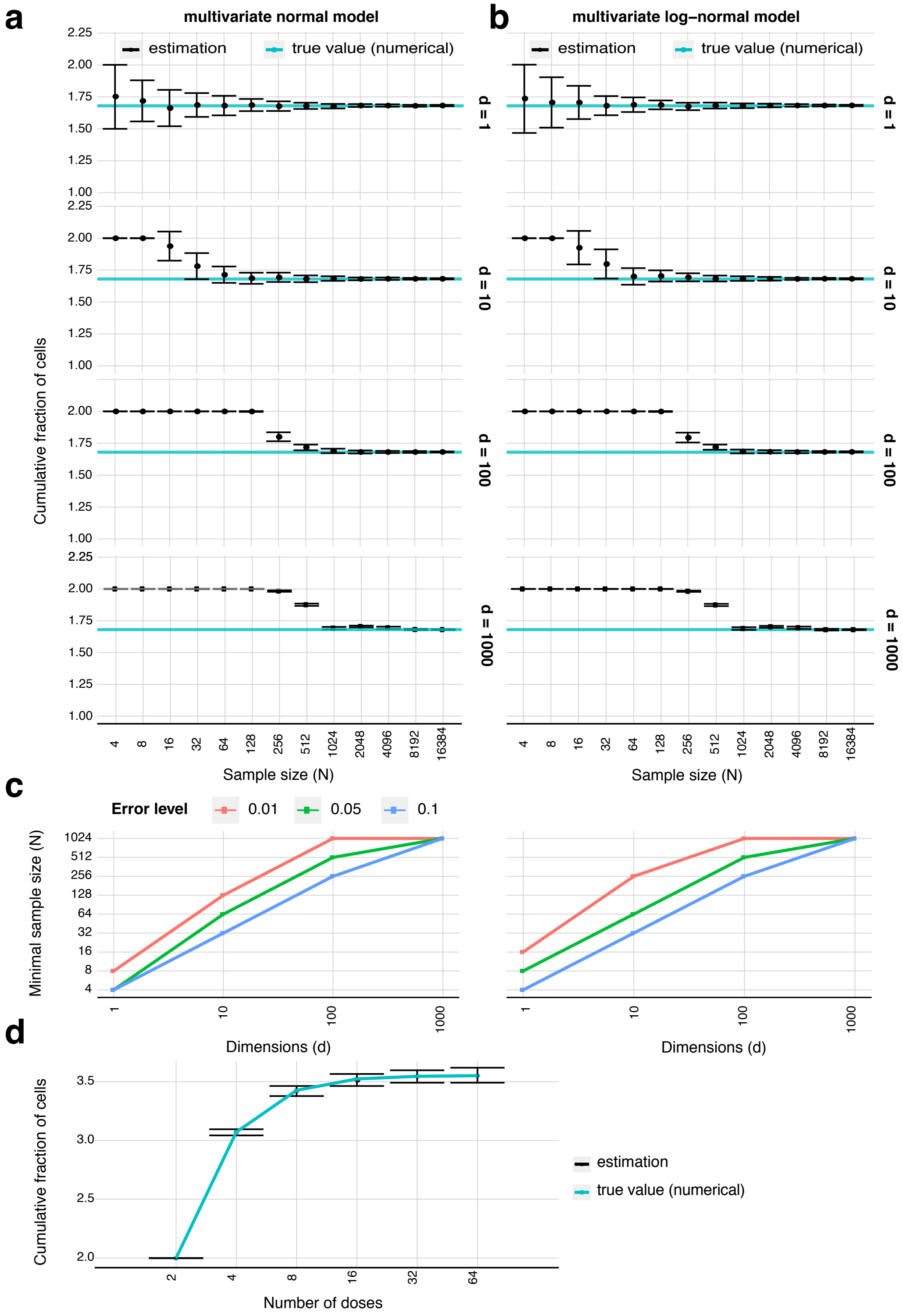

Supplementary Figure 17. Statistical properties of FRA estimation.

a Estimates of the cumulative fraction of cells compared with the exact, numerically computed, value, as a function of the sample size, N, for four different dimensions, d, of the normal model.

b The same as in **a** but for log-normal model.

c Sample size required for estimation with accuracy of 10% (blue), 5% (green), 1% (red) for normal (left column) and log-normal (right column) models computed based on estimates presented in **a** and **b**.

d Estimates of the cumulative fraction of cells for the graded model described in section 3.1 of Supplementary Notes for different number of doses and compared with the true value computed numerically.

Technical details: Data are presented as mean values from 128 repeated samplings +/- standard deviation.
