# Supplementary material for: Fractional response analysis reveals logarithmic cytokine responses in cellular populations
Source: Nat Commun. 2021 Jul 7;12:4175. doi: 10.1038/s41467-021-24449-2 (PMC8263596; doi:10.1038/s41467-021-24449-2)
Supplement: Supplementary file 3 — Reporting Summary [file 41467_2021_24449_MOESM3_ESM.pdf]

nature research

Corresponding author(s): Michał Komorowski

Last updated by author(s): May 24, 2021

Reporting Summary

Nature Research wishes to improve the reproducibility of the work that we publish. This form provides structure for consistency and transparency in reporting. For further information on Nature Research policies, see our Editorial Policies and the Editorial Policy Checklist.

Statistics

For all statistical analyses, confirm that the following items are present in the figure legend, table legend, main text, or Methods section.

n/a	Confirmed	
☐	☒	The exact sample size (n) for each experimental group/condition, given as a discrete number and unit of measurement
☐	☒	A statement on whether measurements were taken from distinct samples or whether the same sample was measured repeatedly
☒	☐	The statistical test(s) used AND whether they are one- or two-sided<br>*Only common tests should be described solely by name; describe more complex techniques in the Methods section.*
☐	☒	A description of all covariates tested
☒	☐	A description of any assumptions or corrections, such as tests of normality and adjustment for multiple comparisons
☐	☒	A full description of the statistical parameters including central tendency (e.g. means) or other basic estimates (e.g. regression coefficient) AND variation (e.g. standard deviation) or associated estimates of uncertainty (e.g. confidence intervals)
☒	☐	For null hypothesis testing, the test statistic (e.g. F, t, r) with confidence intervals, effect sizes, degrees of freedom and P value noted<br>*Give P values as exact values whenever suitable.*
☒	☐	For Bayesian analysis, information on the choice of priors and Markov chain Monte Carlo settings
☒	☐	For hierarchical and complex designs, identification of the appropriate level for tests and full reporting of outcomes
☒	☐	Estimates of effect sizes (e.g. Cohen's d, Pearson's r), indicating how they were calculated

Our web collection on statistics for biologists contains articles on many of the points above.

Software and code

Policy information about availability of computer code

Data collection	Cell Profiler v 2.1.1, R v 3.5.1, Helios CyTOF Software v6.7
Data analysis	R v 3.5.1, imageJ 1.48v, CATALYST v1.5.3.23, cytofCore v0.4, flowCore v1.46.2, Cytobank v6.2, FRA R-package https://github.com/sysbiosig/FRA (https://doi.org/10.5281/zenodo.4818586).

For manuscripts utilizing custom algorithms or software that are central to the research but not yet described in published literature, software must be made available to editors and reviewers. We strongly encourage code deposition in a community repository (e.g. GitHub). See the Nature Research guidelines for submitting code & software for further information.

Data

Policy information about availability of data

All manuscripts must include a data availability statement. This statement should provide the following information, where applicable:
- Accession codes, unique identifiers, or web links for publicly available datasets
- A list of figures that have associated raw data
- A description of any restrictions on data availability

Data generated during the study is available in the public repository: https://github.com/sysbiosig/FRA/ as well as in the open access repository, Zenodo, https://doi.org/10.5281/zenodo.4835622.

Field-specific reporting

Please select the one below that is the best fit for your research. If you are not sure, read the appropriate sections before making your selection.

☒ Life sciences ☐ Behavioural & social sciences ☐ Ecological, evolutionary & environmental sciences

For a reference copy of the document with all sections, see nature.com/documents/nr-reporting-summary-flat.pdf

Life sciences study design

All studies must disclose on these points even when the disclosure is negative.

Sample size	Experiments were performed with at least two biological replicates. In all experiments at least 200 cells were collected per sample. As examined in the Supplementary information a cell number exceeding by order of magnitude the number of measured output variables ensures accurate estimation within FRA.
Data exclusions	No data were excluded, except for CyTOF gating as described in Methods. Precisely, different immune cell subpopulations were gated from single, live, CD45+ cells as shown in Supplementary Figure 2. The criteria for subpopulation assignment were predetermined with specific threshold set after visual inspection of data as explained in Supplementary Figure 2.
Replication	At least two biological replicates were performed. For microscopy experiments each biological experiment involved two technical replicates. All replication attempts were successful, with replicates yielding the same conclusions as described in the paper.
Randomization	Not relevant. This study did not involve experimental grouping.
Blinding	Not relevant. This study did not involve experimental grouping.

Reporting for specific materials, systems and methods

We require information from authors about some types of materials, experimental systems and methods used in many studies. Here, indicate whether each material, system or method listed is relevant to your study. If you are not sure if a list item applies to your research, read the appropriate section before selecting a response.

Materials & experimental systems

n/a	Involved in the study	
☐	☒	Antibodies
☐	☒	Eukaryotic cell lines
☒	☐	Palaeontology and archaeology
☒	☐	Animals and other organisms
☐	☒	Human research participants
☒	☐	Clinical data
☒	☐	Dual use research of concern

Methods

n/a	Involved in the study	
☒	☐	ChIP-seq
☐	☒	Flow cytometry
☒	☐	MRI-based neuroimaging

Antibodies

Antibodies used	CD14 (TUK4) Qdot655 (Thermo Fisher, #Q10056), CD45 (HI30) 89Y (Fluidigm, #3089003B), CD11c (Bu15) 141Pr (Biolegend, #337221), CD11b (ICRF44) 142Nd (Biolegend, #301337), CD45RA (HI100) 143Nd (Biolegend, #304143), HLA-DR (L243) 144Nd (Biolegend, #307651), CD4 (RPA-T4) 145Nd (Biolegend, #300541), CD19 (HIB19) 146Nd (Biolegend, #302247), CD20 (2H7) 147Sm (Biolegend, #302343), CCR6 (G034E3) 148Nd (Biolegend, #353427), CD56 (NCAM16.2) 149Sm (Fluidigm, #3149021B), p-STAT5 (47) 150Nd (Fluidigm, #3150005A), CD45RO (UCHL1) 151Eu (Biolegend, #304239), CD27 (O323) 152Sm (Biolegend, #302839), p-STAT1 (4a) 153Eu (Fluidigm, #3153005A), CD1c (L161) 154Sm (Biolegend, #331502), CD123 (6H6) 155Gd (Biolegend, #306027), p-p38 (D3F9) 156Gd (Fluidigm, #3156002A), p-STAT3 (4/P-Stat3) 158Gd (Fluidigm, #3158005A), p-MAPKAPK2 (27B7) 159Tb (Fluidigm, #3159010A), CD3 (UCHT1) 160Gd (Biolegend, #300443), DNGR1 (8F9) 161Dy (Fluidigm, #3161018B), IFNAR2 (polyclonal) 162Dy (Abcam, #ab56070), STAT1 (246523) 163Dy (Bio-Techne, #MAB1490), IFNAR1 (EP899Y) 164Dy (Abcam, #ab213331), CD161 (HP-3G10) 165Ho (Biolegend, #339919), p-NFkBp65 (K10x) 166Er (Fluidigm, #3166006A), CCR7 (G043H7) 167Er (Fluidigm, #3167009A), p-STAT6 (18/P-Stat6) 168Er (Fluidigm, #3168012A), CD24 (ML5) 169Tm (Fluidigm, #3169004B), CD141 (M80) 170Er (Biolegend, #344102), p-ERK1/2 (D13.14.4E) 171Yb (Fluidigm, #3171010A), CD38 (HIT2) 172Yb (Fluidigm, #3172007B), STAT3 (124H6) 173Yb (Fluidigm, #3173003A), p-STAT4 (38/p-Stat4) 174Yb (Fluidigm, #3174005A), CCR4 (L291H4) 175Lu (Fluidigm, #3175035A), CXCR3 (G025H7) 176Yb (Biolegend, #353733), CD8 (RPA-T8) 198Pt (Biolegend, #301053), CD16 (3G8) 209Bi (Fluidigm, #3209002B), Fc Receptor Binding Inhibitor Antibody (eBioscience, #14-9161-73), phospho-STAT1 (Tyr701) (Cell Signaling, #9167), phospho-STAT3 (Tyr705) (Cell Signaling, #4113), Donkey anti-Rabbit IgG (H+L) Highly Cross-Adsorbed Secondary Antibody, Alexa Fluor 488 (Life Technologies, #A-21206), Donkey anti-Mouse IgG (H+L) Highly Cross-Adsorbed Secondary Antibody, Alexa Fluor 555 (Life Technologies, #A-31570).
Validation	According to the manufacturer's website, anti-phospho-STAT1 (Tyr701) (Cell Signaling, #9167), is quality tested by

Validation	immunofluorescent staining with microscopic analysis. This antibody was also validated through western blotting with STAT1 knockout MEFs.<br><br>According to the manufacturer's website, anti-phospho-STAT3 (Tyr705) (Cell Signaling, #4113), is quality tested by immunofluorescent staining with microscopic analysis.<br><br>According to the manufacturer's website, Donkey anti-Rabbit IgG (H+L) Highly Cross-Adsorbed Secondary Antibody, Alexa Fluor 488 (Life Technologies, #A-21206), is quality tested by immunofluorescent staining with microscopic analysis.<br><br>According to the manufacturer's website, Donkey anti-Mouse IgG (H+L) Highly Cross-Adsorbed Secondary Antibody, Alexa Fluor 555 (Life Technologies, #A-31570), is quality tested by immunofluorescent staining with microscopic analysis.

Eukaryotic cell lines

Policy information about cell lines

Cell line source(s)	U937 cells - ATCC.<br>NIH/3T3 - kind gift from prof. S. Tay, which were preciously generated in the following way: p65-/- mouse embryonic fibroblasts (3T3) were generated by the Baltimore group as described in [1]. p65-/- mouse fibroblast (3T3) cells were subsequently used by Lee et al. [2] to create a reporter cell line expressing the fluorescent fusion protein p65–DsRed under control of the endogenous mouse p65 promoter.<br><br>[1] Beg, Amer A., et al. "Embryonic lethality and liver degeneration in mice lacking the RelA component of NF-κB." Nature 376.6536 (1995): 167-170.<br><br>[2] Lee, Timothy K., et al. "A noisy paracrine signal determines the cellular NF-κB response to lipopolysaccharide." Science signaling 2.93 (2009): ra65-ra65.
Authentication	None of the cell lines used were authenticated upon deliver from the source.
Mycoplasma contamination	All cell lines were tested negative for mycoplasma contamination.
Commonly misidentified lines<br>(See ICLAC register)	To the best of our knowledge neither of the used cell lines can be classified as commonly misidentified.

Human research participants

Policy information about studies involving human research participants

Population characteristics	Participants were aged 25 - 40, male or female, and self reported as feeling healthy.
Recruitment	Healthy, adult volunteers were recruited by email from amongst the staff and students of the Weatherall Institute for Molecular Medicine. Individuals were asked not to volunteer if they believed they may have had a recent infection, with the intention of excluding individuals who may have elevated levels of type I interferons. As similar quantifications of cell-to-cell heterogeneity structure were not performed before it is not possible to predict how selection biases could impact the results. However, given that cell-line experiments yield similar conclusion the bias appears to be negligible.
Ethics oversight	This work was carried out in accordance with the EU Directive 2004/23/EC and the UK Human Tissue Act 2004 (HTA), under the HTA licence (number 12433) of the Weatherall Institute of Molecular Medicine. Informed consent was obtained and the samples were fully anonymised.

Note that full information on the approval of the study protocol must also be provided in the manuscript.

Flow Cytometry

Plots

Confirm that:

☒ The axis labels state the marker and fluorochrome used (e.g. CD4-FITC).

☒ The axis scales are clearly visible. Include numbers along axes only for bottom left plot of group (a 'group' is an analysis of identical markers).

☒ All plots are contour plots with outliers or pseudocolor plots.

☒ A numerical value for number of cells or percentage (with statistics) is provided.

Methodology

Sample preparation	PBMCs were isolated from the peripheral blood of healthy adult donors using Lymphoprep (Stemcell Technologies), according to the manufacturer's instructions.<br>Cells were washed in serum-free RPMI then resuspended at 10^7 cells/mL in serum-free RPMI containing 0.5 mM Cell-ID Cisplatin (Fluidigm) and incubated at 37°C for 5 min. Cells were washed with RPMI containing 10% (v/v) FCS (Sigma) and 2 mM L-Glutamine (R10), centrifuging at 300 x g for 5 min before being resuspended to 6 x 10^7 cells/mL in R10 and rested at

37◦C for 15 min. 50 mL of cells (3 x 10^6 cells) were transferred to 15 mL falcon tubes for stimulation and antibody staining. Antibodies are listed in Supplementary Table 1. Staining for CD14, CCR6, CD56, CD45RO, CD27, CCR7, CCR4 and CXCR3 was done before stimulation/fixation for 30 min in R10 at 37◦C.
Cells were stimulated with 0, 25, 250, 2500 or 25000 U/mL recombinant human IFN-α2a (PBL Assay Science, #11100-1) diluted in R10 for 15 min at 37◦C.
After washing with 5 mL cold Maxpar PBS (Fluidigm), cells were fixed with 1 X Maxpar Fix I Buffer (Fluidigm) for 10 min at RT before being washed with 1.5 mL Maxpar Cell Staining Buffer (CSB, Fluidigm). All centrifugation steps after this point were at 800 x g for 5 min. Cells were barcoded using Cell-ID 20-Plex Pd Barcoding Kit (Fluidigm), according to the manufacturer's instructions, and washed twice with CSB before samples were pooled and counted. All further steps were performed on the pooled cells.
Fc receptors were blocked using Fc Receptor Binding Inhibitor Antibody (eBioscience, #14-9161-73) diluted 1:10 in CSB for 10 min at RT. Surface antibody staining mixture was added directly to the blocking solution and incubated for 30 min at RT. Cells were washed twice with CSB, resuspended in ice-cold methanol and stored at -80◦C overnight.
After washing twice with CSB, cells were stained with intracellular antibody staining mixture for 30 min at RT before two further washes in CSB. Cells were resuspended in 1.6% (v/v) formaldehyde (Pierce, #28906) diluted in Maxpar PBS and incubated for 10 min at RT. Cells were resuspended in 125 mM Cell-ID Intercalator (Fluidigm) diluted in Maxpar Fix and Perm Buffer (Fluidigm) and incubated overnight at 4◦C. Compensation beads (OneComp eBeads Compensation Beads, Invitrogen, #01-1111-42) stained with 1 mL of each antibody were also prepared.
The next day, cells and compensation beads were washed twice with CSB and twice with Maxpar water (Fluidigm), mixed with a 1:10 volume EQ Four Element Calibration Beads (Fluidigm) before acquisition on a Helios Mass Cytometer (Fluidigm) using the HT injector.

Instrument

Fluidigm Helios CyTOF System

Software

Data were collected using the software on the instrument (Helios CyTOF Software v6.7). Data were also normalized, randomized and multiple .fcs files concatenated using this software. Compensation and debarcoding was performed using the CATALYST package (v1.5.3.23). .fcs files were rewritten using the updatePanel function of the cytofCore package (v0.4) before being imported into Cytobank (v6.2) for gating to single, live, CD45+ cells.

Cell population abundance

At least 2500 cells were measured per condition.

Gating strategy

Cells were first gated as negative for 140Ce (EQ beads) and positive for 191Ir (Iridium, DNA intercalator). Intact cells were identified as the central population of 191Ir+ 193+ events. Single cells were then gated as Event_length-low and live cells as 194Pt- 195Pt- (Cisplatin, viability reagent). CD45+ cells were gated and exported as a new .fcs file for each sample.

☒ Tick this box to confirm that a figure exemplifying the gating strategy is provided in the Supplementary Information.
